# Nutritional composition, bioactive compounds and antioxidant potentiality of some indigenous vegetables consumed in Bangladesh

**DOI:** 10.1038/s41598-024-78625-7

**Published:** 2024-11-12

**Authors:** Khurshida Jahan Mila, Jahidul Hassan, Md. Fakhrul Hasan, Alanoud T. Alfagham, Liakat Ali, Md. Saiful Islam, Md. Zubayer, Joydeb Gomasta, Yukio Ozaki, Manzer H. Siddiqui, Farhan Khalid, Md. Ferdous Mondal

**Affiliations:** 1 Department of Horticulture, Faculty of Agriculture, Bangabandhu Sheikh Mujibur Rahman Agricultural University, Gazipur, 1706 Bangladesh; 2 Department of Botany and Microbiology, College of Science, King Saud University, Riyadh, 11451 Saudi Arabia; 3 Department of Genetics and Plant Breeding, Faculty of Agriculture, Bangabandhu Sheikh Mujibur Rahman Agricultural University, Gazipur, 1706 Bangladesh; 4 Laboratory of Horticultural Science, Faculty of Agriculture, Kyushu University, Fukuoka, 819- 0395 Japan; 5 Faculty of Agriculture and Environment, The Islamia University of Bahawalpur, Bahawalpur, 63100 Punjab Pakistan; 6 College of Agriculture, International University of Business, Agriculture and Technology, Dhaka, 1230 Bangladesh

**Keywords:** Antioxidant activity, Indigenous vegetables, Nutritional value, Minerals, Biochemistry, Plant sciences

## Abstract

Food and nutrition security are essential for accomplishing sustainable development goals, and a growing population requires various food sources to address hunger and nutrition. Indigenous vegetables are nutritious, healthy, and adaptable to local conditions, making them a potential food and nutrition security resource. Thus, this study aimed to evaluate the nutritional richness of indigenous vegetables by comparing the nutritional content of commonly grown indigenous vegetables like *Chenopodium album* (Bathua), *Coccinia grandis* (Telakucha), *Amaranthus viridis* (Shaknotey), *Moringa oleifera* (Shojne), *Alternanthera philoxeroides* (Malancha), and *Xanthium strumarium* (Ghagra) to BARI Lalshak-1 (*Amaranthus tricolor*). Shojne violet had the highest lightness (L*), directions (b*), and chroma (c*) of 43.50, 23.69, and 25.02, respectively and telakucha had the highest luminosity (h°) of 114.11. Shaknotey had more moisture (88.97 %) than bathua green (66.62 %) or ghagra (72.55 %). Ghagra had the most chlorophyll a (1.11 mg/g), b (0.65 mg/g), total chlorophyll (2.04 mg/g), ascorbic acid (22.0 mg/100 g), and flavonoids (50.1 mgQE/100 g). Bathua red had significant levels of anthocyanin, carotenoids, and strongest antioxidant activity (IC50 value): 42.9 µg/g, 0.24 mg/g, and 12.4 µg/mL, respectively. Shojne green and telakucha were found rich in phenolic content (136.0 mgGAE/100 g) and β-carotene (2.05 mg/100 g), respectively. Moreover, the studied indigenous vegetables were rich in minerals, potassium, calcium, magnesium, and iron, with a very high K content (49.4–79.4 mg/g). Correlation matrix and PCA showed that ghagra, shojne, and bathua species were nutritionally superior to others. Therefore, the current findings suggest that ghara, shojne and bathua can become a nutrient-rich indigenous vegetable and improve human nutrition.

**Supplementary Information:**

The online version contains supplementary material available at 10.1038/s41598-024-78625-7.

## Introduction

Zero Hunger (SDG-2) and Good Health and Wellbeing (SDG-3) have perceived considerable attention in recent years among the 17 Sustainable Development Goals (SDGs) due to the alarming statistic in 2020 that 811 million individuals experienced hunger and a quarter of the global population lacked access to sufficient nutritious food in 2019^1^. The COVID-19 pandemic in the recent past has exacerbated this situation, with forecasts for 2030 suggesting that the current efforts are far from sufficient to eradicate malnutrition within the next decade^1^. Furthermore, food systems encounter significant obstacles due to environmental degradation, loss of resources, influence of climate change, emissions of greenhouse gases and population expansion. These intensifies agricultural vulnerabilities causing unfavorable conditions, which creates critical condition for major crop production^2^. Indigenous vegetables (IVs) have drawn considerable scientific attention because of their potential to address nutrition and hunger issues in this climate change scenario^3^. Indigenous vegetables (sometimes called ‘neglected’, ‘minor’, ‘orphan’, ‘promising’, ‘coarse’ or ‘little-used’) mostly belong to the non-staple foods^2^ and well adapted to marginal and stress prone conditions that often can be grown with minimal external inputs^4,5^. These underutilized vegetables have high amount of nutrients^6,7^, possess health-protecting properties^8^ and adapted to local environments that are unsuitable for other crops where they provide sustainable productions^9^ but not attain the same level of domestication, global recognition, conservation and adequate research attention as popular vegetables like tomato or eggplant^10,11^. Several wild edible plants are traditionally consumed along with staple foods and also used in treating certain medical conditions^12^, especially in rural areas and a few urban communities in Bangladesh. These traditional IVs have the potential to reduce the excessive dependence of mainstream agriculture on a few major crops and would be Future Smart Foods (FSFs) with fulfilling the criteria of nutrient dense, economically viable, climate resilient and locally adaptable or available^13^. Ninety vegetables are reported to be grown in Bangladesh, nineteen of which are considered as major indigenous vegetables (IVs) due to their origin, growing methods and consumption habits while others are minor IVs that are grown as volunteer plants or weeds with other crops^14^. The weed survey indicated that 19 out of 95 weed species are often consumed as vegetables across the country. Among these indigenous vegetables (IVs) bathua, telakucha, shaknotey, shojne, malancha and ghagra are widely known as weed but nowadays these are considered as vegetables because of their taste and consumers’ acceptability.

Bathua (*C. album*) is referred to as lamb’s quarters (in English) of the family Chenopodiaceae is also known as fat-hen, chakvit^15^ which commonly found along roadside paths, bushes and gardens where it grows as erect annual herb producing angular stems, ribbed with longitudinal dark green or red streaks. Telakucha or Ivy gourd (*C. grandis)* belongs to the Cucurbitaceae family^16^ is found in tropical Asia and grows abundantly in Bangladesh. It is a climbing perennial herb, with soft stem and fleshy leaves, growing throughout bushes and gardens especially in warmer and humid climatic conditions. Shaknotey (*A. viridis*) referred as slender amaranth or green amaranth belongs to the family Amaranthaceae, is an annual herb with an upright, light green stem that grows to about 60–80 cm in height^17^. Shojne (*M. oleifera)* is commonly known as drum stick tree belongs to the family Moringaceae^18^, is a fast-growing drought resistant tree, also called “miracle tree” or “tree of life” is native to Bangladesh^19^. Malancha (*A. philoxeroides)* commonly known as “Alligator weed” in English, locally known as ‘Henchi Shak’ in Bangladesh^20^, is a leafy vegetable from the family Amaranthaceae which leaves are consumed as a vegetable. Ghagra (*X. strumarium*) belongs to the family Asteraceae; is an annual upright herb with rough hairs, attaining a height of 150 cm, the stem is black or brown-spotted and the leaves are hairy in both parts, triangular almost look like brinjal leaf. Local people collect these vegetables from adjacent ecosystems such as nearby their homes, fallow lands, fields, roadsides, and gardens, where they flourish as prevalent weeds. Although these vegetables are frequently consumed in different areas, there is still a lack of information regarding their nutritional composition that hinders their potential as important source of dietary nutrients. Identifying and quantifying important compounds in these vegetables to evaluate their nutritional composition and potential anti-nutrients (compounds that interfere with the absorption of nutrients, for instance, oxalate, phytate) will provide preliminary knowledge on their nutritional activity. Therefore, this experiment was conducted to determine the nutritional composition, minerals, antioxidant, anthocyanin and phytochemical constituents among these selected indigenous vegetables and comparing their nutritional components with BARI lalshak-1 (*A. tricolor*) because of its wide acceptance and registered cultivar as leafy vegetable, color pigmentation and well documented nutraceuticals properties^17,21,22^.

## Materials and methods

### Study approach

The study was carried out from September, 2022 to December, 2023 at the Horticultural research field, Nursery and Tissue Culture Laboratory, Department of Horticulture, Bangabandhu Sheikh Mujibur Rahman Agricultural University, Salna, Gazipur-1706, Bangladesh. The experimental site is located at the center of the Madhupur tract about 24.02° N latitude and 90.23° E longitude at 8.40 m above sea level.

**Fig. 1:**
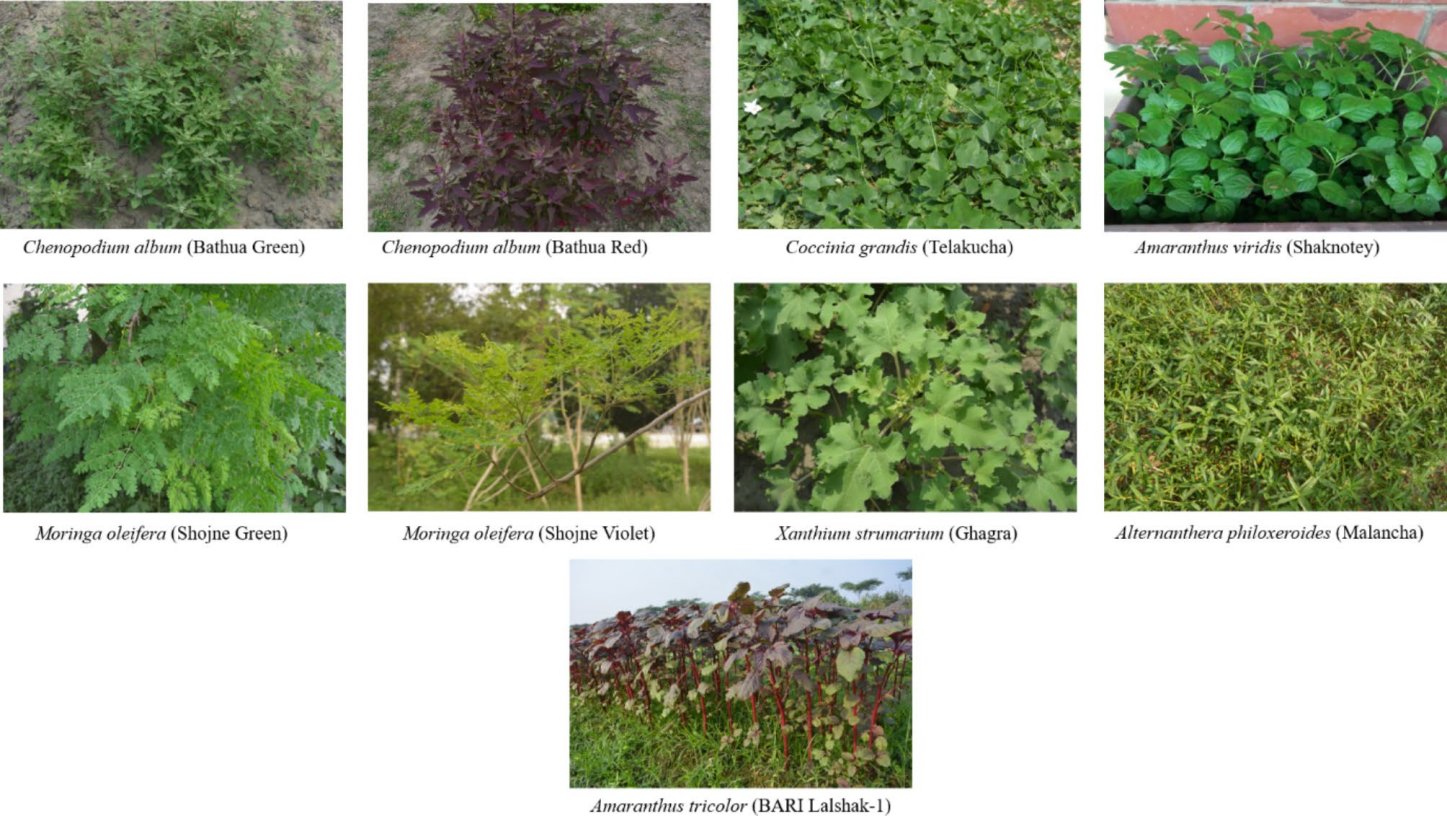
Eight indigenous and one cultivable vegetable used for analysis.

The eight (8) different types of indigenous vegetables (IVs) such as bathua—*C. album* (red and green species), telakucha (*C. grandis*), shaknotey (*A. viridis*), shojne (*M. oleifera* -violet and green), Malancha (*A. philoxeroides* (Mart.)) and ghagra (*X. strumarium*) leaves were used as plant materials and BARI lalshak-1 (*A. tricolor)* as control (Fig. 1). The fresh and fully expanded young leaves were collected from the research field and nursery of the Department of Horticulture, BSMRAU. The collected materials were chopped/blend by using scissor/blender and used for different nutritional analyses. Representative samples were allowed to dry for 72 h in an oven at 70 ℃. The dried samples were crushed into powder using grinder and stored in air-tight zipper bag until used for further analyses.

### Color estimation

The colors of the studied vegetables were measured using a bench-top spectrophotometer (CR-5; Konica Minolta). The fresh and fully expanded young leaves from the selected vegetables were randomly collected, then measured at their mid-point of adaxial surface. The color change was determined as L* indicates the darkness and lightness of color and ranges from 0 to 100 (L* = 0 means black and L* = 100 means white). Color parameters a* and b* extend from − 60 to + 60 [− a* = green and + a* = red; − b* = blue and + b* = yellow]. A white standard plate was used to calibrate the spectrometer before use to ensure the accuracy of the data. The hue angle (h°) is expressed in degrees from 0° to 360° (0° = red, 90° = yellow, 180° = green, and 360° = blue)^23^ The hue angle and Chroma (C) were calculated by following equations 

1 $$h=arctan\left( {\frac{{b*}}{{a*}}} \right)$$

2 $$C=\sqrt {\left( {{\text{~}}{a^{*2}}+{\text{~}}{b^{*2}}} \right)}$$

### Nutritional composition analyses

#### Determination of β-carotene

β-carotene was estimated spectrophotometrically according to the previous procedure^24–26^. At first, 10 mL mixed solution of acetone: hexane (4:6) (v/v) was added to each 1 gm of leaf sample, grinded with mortar pestle and the supernatant was filtered with Whatman filter paper in a test tube followed by sealing the tube with aluminium foil paper (this process was followed for every replication). Optical density of the sample was measured by spectrophotometer (PD-303 UV Spectrophotometer; APEL Co.) at 663 nm, 645 nm, 505 nm, and 453 nm.

The following formula was used for the calculation of β-carotene content (mg/100 g)

3 $${\text{b}} - {\text{carotene }}\left( {{\text{mg}}/{\text{1}}00{\text{ g}}} \right)\, = \,0.{\text{216 }}\left( {{\text{OD663}}} \right)\, + \,0.{\text{452 }}\left( {{\text{OD453}}} \right)\; - \;{\text{1}}.{\text{22 }}\left( {{\text{OD645}}} \right)\; - \;0.{\text{3}}0{\text{4 }}\left( {{\text{OD5}}0{\text{5}}} \right)$$

Where, OD = Optical density.

#### Determination of chlorophyll and carotenoids content

For the determination of chlorophyll a, chlorophyll b, total chlorophyll and carotenoid, 100 mg of plant sample were taken in a glass vial and 5 ml 80% acetone was added. The vials were made airtight and kept at 4 °C in the dark for 24 h. After extraction, the separated plant extracts were taken to measure the absorbance through a spectrophotometer at 663, 646, and 470 nm wavelengths respectively. 80% acetone was used as blank^24,26^.

The quantification was done according to the formula.

$${\text{chla}}(\upmu {\text{g}}/{\text{mL}})\, = \,{\text{12}}.{\text{21 }}\left( {{\text{Absorbance at 663 nm}}} \right)\; - \,{\text{2}}.{\text{81 }}\left( {{\text{Absorbance at 646 nm}}} \right)$$

$${\text{chlb}}(\upmu {\text{g}}/{\text{mL}})\, = \,{\text{2}}0.{\text{13 }}\left( {{\text{Absorbance at 646 nm}}} \right)\; - \,{\text{5}}.0{\text{3 }}\left( {{\text{Absorbance at 663 nm}}} \right)$$

$${\text{Total chl }}(\upmu {\text{g}}/{\text{mL}})\, = \,{\text{2}}0.{\text{2 }}\left( {{\text{Absorbance at 646 nm}}} \right)\, + \,{\text{8}}.0{\text{2 }}\left( {{\text{Absorbance at 663 nm}}} \right)$$

$${\text{Carotenoids }}(\mu {\text{g m}}{{\text{L}}^{ - \,{\text{1}}}}){\text{ }}={\text{ }}[{\text{1}}000{\text{ }}\left( {{\text{Absorbance at 47}}0{\text{ nm}}} \right) - \,{\text{3}}.{\text{27 }}\left( {{\text{chla}}} \right) - {\text{1}}0{\text{4 }}\left( {{\text{chlb}}} \right)]{\text{ }}/{\text{ 229}}$$

To express the amounts as mg/g: $\mu {\text{g}}/{\text{mL}} \times {\text{V}}/{\text{ }}({\text{1}}000 \times {\text{W}})$

Where,

V = Volume of acetone used (mL). W = Weight of leaf sample (grams).

#### Determination of ascorbic acid content

Ascorbic acid content of fresh indigenous vegetables was determined using the titration method^24,26,27^ with some modifications. Sample was prepared by blending 20 g of fresh leaves with distilled water and centrifuge at 4 °C for 20 min at a speed of 6000 rpm with a centrifuge machine (MPW-260R). The supernatant liquid was collected in a test tube and covered with foil paper. For ascorbic acid estimation, 5 mL of the prepared sample extract was taken in a 50 mL conical flask and added 5 mL of 5% KI, 2 mL of Glacial acetic acid and 2 mL of 2% starch solution to the extract. Then, it was titrated with 0.001 N (potassium iodate) KIO_3_ solution.

The ascorbic acid (mg/100 g of the sample solution) was estimated by using the following equation:

4 $${\text{Ascorbic acid content }}=\frac{{{\text{T}} \times {\text{F}} \times {\text{V}} \times 100}}{{{\text{v}} \times {\text{W}}}}$$

Where,

T = titrated volume of 0.001 N KIO_3_(mL). F = 0.088 mg of ascorbic acid per ml of 0.001 N KIO_3_. V = total volume of sample extracted (mL). v = volume of the extract (mL) titrated with 0.001 N KIO_3_.

#### Determination of anthocyanin

Anthocyanin was estimated spectrophotometrically according to the previous procedure^24,26,28^. At first, 1.0 g sample leaves from each replication were taken in an ice-cold glass vial. 5 mL extraction solution (Methanol: 6 M HCL: Water = 70:7:23 (v/v/v) was added to each vegetable leaves vial and only extraction solution in another vial. This extraction solution without sample leaves was used as blank. Then vials were made airtight and kept at 4 °C in the dark for 24 h. After 24 h, 2 mL solution from the vial was taken into a centrifuge tube and 2 mL distilled water was added in each tube. Then, 2 mL chloroform was added to each vial to separate anthocyanin (insoluble in chloroform) from chlorophylls. After that, mixtures were centrifugated for 15 min at 5000 rpm at 4 °C. After centrifugation, 3 mL of the top layer (containing anthocyanin) was taken in a glass cuvette. Then, absorbance was measured at 530 nm wavelength.

The total anthocyanin content (TAC) was calculated using the equation:

5 $${\text{TAC }} = \frac{{Abs \times {\text{MW}} \times {\text{V}} \times {\text{DF}} \times {\text{CF}}1 \times {\text{CF}}2}}{{\varepsilon \times {\text{l}} \times {\text{W}}}}$$

Where:

TAC = total anthocyanin content expressed as µg/g. Abs = absorbance at 530 nm. MW = molecular weight of cyanidin-3-glucoside (449 g/moL). DF = dilution factor (1). l = path length (1 cm). CF1 = conversion factor 1 (10^6^ µg/g). CF2 = conversion factor 2 (1 L/1000 mL). ε = molar extinction coefficient of cyanidin-3-glucoside (30,000 L/molcm). W = Used sample weight (g).

#### Determination of minerals and moisture content (MC) (%)

The mineral content (K, Na, Ca, Mg) was determined by using AAS (atomic absorption spectrophotometer), following the previous procedure^24,29^. In this aspect, 10 g of dried sample was grounded for preparing powder and 0.5 g sample powder was taken in a test tube then added 5 mL HNO_3_ and digested at 60 °C temperature for 30 min. Then added 2 mL HClO_4_ (perchloric acid) and further digested at 150 °C for 60 min through digestion chamber. The last portion of the digestion process was filtered with Whatman 42 (2.5 μm particle retention) filter paper and added di-ionized water up to the final volume of 50 mL in a 50 mL volumetric flask. For determining the mineral content, 5 mL sample extract was taken in a 50 mL volumetric flask and filled up to 50 mL with di-ionized water. Afterwards, the concentration of K, Na, Ca, Fe and Mg was measured through AAS (atomic absorption spectrophotometer; model-PinAAcl 900 H; PerkinElmer).

The mineral concentration was measured using the following formula

6 $${\text{Mineral }}\left( {{\text{mg}}/{\text{kg}}} \right)={\text{~~~}}\frac{{{\text{sample~reading}} \times {\text{final~volume}} \times {\text{dilution~factor}}}}{{{\text{sample~weight}}}}$$

The fresh vegetables were used for measurement of water content. Initially, the fresh weight of the sample recorded and thereafter, dried to a constant mass in an oven at a temperature of 72 °C for 72 h. Then final weight was recorded and percent (%) moisture estimated on the basis of fresh and dry masses of leaves in g by using the following equation.

 

7 $$   {\text{\% ~Moisture}} = \frac{{{\text{Initial~}}\;{\text{weight~}}\left( {\text{g}} \right) - {\text{Final~}}\;{\text{weight~}}\left( {\text{g}} \right)}}{{{\text{Initial}}\;{\text{~Weight~}}\left( {\text{g}} \right)}} \times 100 $$

#### Preparation of methanolic extract used for bio-active compounds estimation

For the preparation of methanolic extract 1 g fresh sample was weighted with electronic precision balanced (Digiscales, Germany), blended and immersed in methanol (25 mL) in a test tube. The test tube was placed in a water bath at 30 °C for two and half h. Then the sample was centrifuged at 6000 rpm for 15 min and the supernatant was filtered using funnel and Whatman 42 filter paper which was stored at 4 °C in a refrigerator. The amount of total bioactive compounds that act as non-enzymatic antioxidants such as phenols, flavonoids and total antioxidant activity was determined from these methanolic extracts.

### Bio-active compound estimation

#### Determination of total phenolic content (TPC) (mg GAE/100 g FW)

The total phenolic content (TPC) from fresh samples of the sample vegetables were estimated using the Folin–Ciocalteu procedure^24,26,30^ with some modification. For the determination of phenolic content, a previously produced methanol extract solution was used. For preparing the stock solution, 5 mL of FC reagent was pipetted in a conical flask and 45 mL of water for preparing 10 times dilution. For the preparation of 7.5% Na_2_CO_3_, 7.5 g of sodium carbonate was added with 100 mL of distilled water in a 100 mL conical flask. For the preparation of gallic acid standard, 100 mg gallic acid powder was weighted and diluted in 100 mL of distilled water. Then different concentration (10, 20, 40, 60, 80, 100, 200 µL) of gallic acid was measured for the calibrations curve. For the estimation of TPC, 0.5 mL of the sample extracts were taken in a test tube. Different concentrations of gallic acid were also added in a test tube. Folin-Ciocalteu reagent (2.5 mL) was added to the sample and the standard solutions, and the reactions stood for 10 min. Then 2 mL of 7.5% sodium carbonate was mixed with the solution and the resultant mixture was incubated at 30 °C for 1 h. The absorbance reading of the sample and the gallic acid standard were measured at 760 nm by using a UV-VIS (PD-303 UV Spectrophotometer; APEL Co.) spectrophotometer against methanol blank as standard. The standard curve was prepared using Microsoft excel using absorbance of gallic acid and TPC readings were measured against the gallic acid standard calibration curves.

The TPC were expressed as mg of gallic acid equivalents per 100 g fresh weight by following the formula as below,

8 $${\text{y}}\,=\,{\text{mx}}\,+\,{\text{c}}$$

Where,

y = Absorbance of samples. x = Total phenol content (TPC).

The value of c and m was found from the regression line plotted for each sample separately.

#### Determination of total flavonoid content (TFC) (mg QE/100 g FW)

Total flavonoid content was determined by aluminum chloride colorimetric method^26,31,32^ with some modifications. Here, a similar methanolic sample extract was used as a working sample previously used for phenol content determination. For total flavonoid content estimation, quercetin was used to make the standard calibration curve. The stock solution of quercetin was prepared by dissolving 10 mg quercetin in 10 mL methanol. Then different concentration (10–100 µg/mL) was measured for calibrations carve of quercetin and added methanol up to the final volume of 1000 µL. For TFC analysis, 100 µL sample extract was taken in the Eppendorf tube and added 400 µL of methanol and different concentration of quercetin also. Each sample extracts and standards of quercetin were separately mixed with 100 µL of 10% (w/v) AlCl_3_ and 100 µL of 1 M potassium acetate. Then it was incubated at room temperature and in dark condition for 40 min followed the measurement of absorbance at 420 nm using a spectrophotometer against the blank.

The outcome data of TFC was calculated from the quercetin standard calibration curve and expressed as mg quercetin equivalent (QE)/100 g fresh weight by following the formula as below,

9 $${\text{y}}\,=\,{\text{mx}}\,+\,{\text{c}}$$

Where,

y = Absorbance of samples. x = Total flavonoid content (TFC).

Value of c and m was found from the regression line plotted for each sample.

#### Determination of total antioxidant activity (TAA)

Antioxidant activity of the fresh sample vegetables was estimated using DPPH radical scavenging assay (RSA). This assay is based on the measurement of the scavenging ability of antioxidants towards the stable radical. It was conducted according to the procedure of^24,26,31,33^ with some modifications where the extraction of 0.5 g powdered sub-sample with 12.5 mL methanol was placed in water bath at 30 °C for 2.5 h and centrifuged at 6000 rpm for 15 min. The supernatants were decanted and filtrated by Whatman filter paper (no. 42) into the test tube covered with foil paper and stored at 4 °C until the samples were analyzed. For preparing the standard calibration curve, ascorbic acid was used. For antioxidant assay each extract and ascorbic acid (2 mg ascorbic acid dissolved in 2.5 mL distilled water and mixed thoroughly) were prepared into several concentration of 20, 40, 80 and 100 µg/mL and added methanol up to 3 mL. Then 1 mL of methanolic DPPH solution (0.004 mg DPPH was added with 100 mL of methanol and mixed properly) was added. Kept the reaction mixture at dark place for 30 min and measured reading at 517 nm against blank by using spectrophotometer.

The scavenging activity was calculated by following formula

10 $$\% {\text{ Radical scavenging activity }}=\frac{{{\text{A}}0 - {\text{A}}1}}{{A0}} \times 100$$

Where,

A_0_ = Absorbance of control (4 mL methanol + 0.5 mL methanolic DPPH solution). A_1_ = Absorbance of sample.

Inhibition concentration (IC_50_) used to indicate antioxidant capacity and was determined from the graph that plotted % radical scavenging activity against concentration of extract for standards and test sample. The values of IC_50_ used in this study were generated from a regression line graph that plotted by % radical scavenging activity of 4 concentrations against 4 concentrations of each extract sample of standard and test sample. IC_50_ can scavenge 50% of DPPH free radical in DPPH free radical scavenging method. As lower IC_50_ value corresponds with a higher antioxidant activity^24,26,34^.

IC_50_ was calculated by following the formula as below

11 $${\text{I}}{{\text{C}}_{{\text{5}}0}}=\frac{{y - b}}{a} \times 100$$

Where,

Y is replaced by 50 in the above equation.

Value of a and b was found from the regression line plotted for each sample separately.

### Statistical analysis

All the recorded data on nutrients, minerals and secondary metabolites of the studied IVs represent the mean values of three technical replications and were subjected to compare by two-way analysis of variance (ANOVA). The mean separation was done following Duncan Multiple Range Test (DMRT) at 5% level of significance (*P* < 0.05). Furthermore, correlation matrix, cluster analysis, were performed to note the interrelationship among the studied variables and the IVs of the study. Afterwards, principal component analysis (PCA) was performed to show the patterns of all the measured correlated nutrient composition, secondary metabolites and minerals in the reduced dimensions of newly obtained factors those were denoted as- Dim1 (PC1), Dim2 (PC2). The dendrogram cluster analysis was performed to sort out the most promising IVs (indigenous vegetables) according to the factor loadings and the contributions of each of the studied dependent variables using different packages (agricolae, facatominer, factoextra, ggplot2, corrplot) of R program (version 4.1.2). All data were reported as the mean value of three determinations ± standard deviation (SD).

## Results

### Color

The color of vegetables underwent a notable variation as indicated in Table 1. The system utilizes L* to quantify the lightness of the color, ranging from white to black. Additionally, a* and b* indicate distinct color directions, with a* ranging from green to red and b* ranging from blue to yellow. Lastly, c* is used to measure the chroma of the color. Table 1 shows that the shojne violet had the lightest L* value (43.50), which was similar to the shojne green (41.88) for their leaves. The control plant lalshak had the darkest L* value of 33.93, followed by telakucha with a value of 34.48 and shaknotey (35.41). Bathua green and ghagra had almost similar L* value of 35.41 and 38.17, respectively. In terms of other color coordinates, the light color a* value varied from 4.77 to 8.26. The highest value of 4.77 was observed in lalshak (control), which represents red color leaves. This value was somehow close to bathua red (0.65, megenta color), but significantly different from the other vegetables. The lowest value of − 8.26, indicating almost white color base of the leaves (pale greenish), was recorded in the shojne green. In addition, the values of b* for the other color directions varied from 23.69 to 6.27. This corresponds to the color spectrum from yellow to purple in a leave. The highest b* value, 23.69, was obtained from shojne violet, which showed a statistically significant difference compared to all other vegetables. Following shojne violet, shojne green had b* value of 20.25, while the control vegetable lalshak had the lowest value of 6.27. Shojne violet exhibited the maximum color saturation, with a C* value of 25.02, which was statistically distinct from the other selected vegetables. Nevertheless, shojne green exhibited the second highest value (21.89), which was statistically comparable to bathua green (19.24), while telakucha and shaknotey had statistically similar value of 13.15 and 13.75, respectively and lalshak (control) had the lowest vividness of color (7.79). However, the decrease in values of a* and the increase in values of b* are associated with the perception of darkness and lightness. Similarly, the highest luminosity (h°) was observed at telakucha (114.11), indicating a brightening of leaves close to a yellow color, statistically close to shojne green (112.11) and bathua green (111.22) followed by malancha (109.52), shojne violet (108.59), ghagra (108.22) and shaknotey (107.39). Whereas, the control vegetable lalshak showed the lowest value of 52.13. Therefore, the values of the parameters accurately depicted the color patterns of the leaves, aligning with the visual observations of the vegetables’ leaves color.

**Table 1:** Colorimetric parameters of different indigenous vegetables.

Vegetables	L*	a*	b*	c*	h°
Bathua Green	38.96 ± 0.13 d	-6.83 ± 0.13 ef	17.78 ± 0.13 c	19.24 ± 0.13 c	111.22 ± 0.13 ab
Bathua Red	36.28 ± 0.51 f	0.65 ± 0.53 b	12.43 ± 0.53 g	12.51 ± 0.53 h	85.12 ± 0.53 c
Telakucha	34.48 ± 0.17 h	-5.34 ± 0.17 cd	11.92 ± 0.17 h	13.15 ± 0.17 g	114.11 ± 0.17 a
Shaknotey	35.41 ± 0.06 g	-4.13 ± 0.06 c	13.22 ± 0.06 f	13.75 ± 0.06 f	107.39 ± 0.06 b
Shojne Violet	43.50 ± 0.06 a	-7.83 ± 0.06 fg	23.69 ± 0.06 a	25.02 ± 0.06 a	108.59 ± 0.06 ab
Shojne Green	41.88 ± 0.05 b	-8.26 ± 0.04 g	20.25 ± 0.06 b	21.89 ± 0.04 b	112.11 ± 0.04 ab
Malancha	40.17 ± 0.03 c	-5.77 ± 0.03 de	16.77 ± 0.03 d	17.74 ± 0.03 d	109.52 ± 0.03 ab
Ghagra	38.17 ± 0.20 e	-5.33 ± 0.20 cd	15.69 ± 0.20 e	16.38 ± 0.20 e	108.22 ± 0.20 ab
Lalshak (Test)	33.93 ± 0.02 h	4.77 ± 0.02 a	6.27 ± 0.02 i	7.79 ± 0.02 i	52.13 ± 0.02 d

[^x^ Color parameters base on CIE (International Commission on Illumination) system for color representation: (L*: Lightness, a*: greenness (−) to redness (+), b*: blue (−) to yellow (+), c*: saturation of the color, h°: huge angle). Data presented as means ± standard deviation in each column followed by different letters are significantly different at *p* < 0.05 as determined by Honestly significant difference test (Tukey’s HSD test) using the R program].

### Nutritional composition of selected indigenous vegetables

#### Chlorophyll (chl) content (mg/g)

**Fig. 2:**
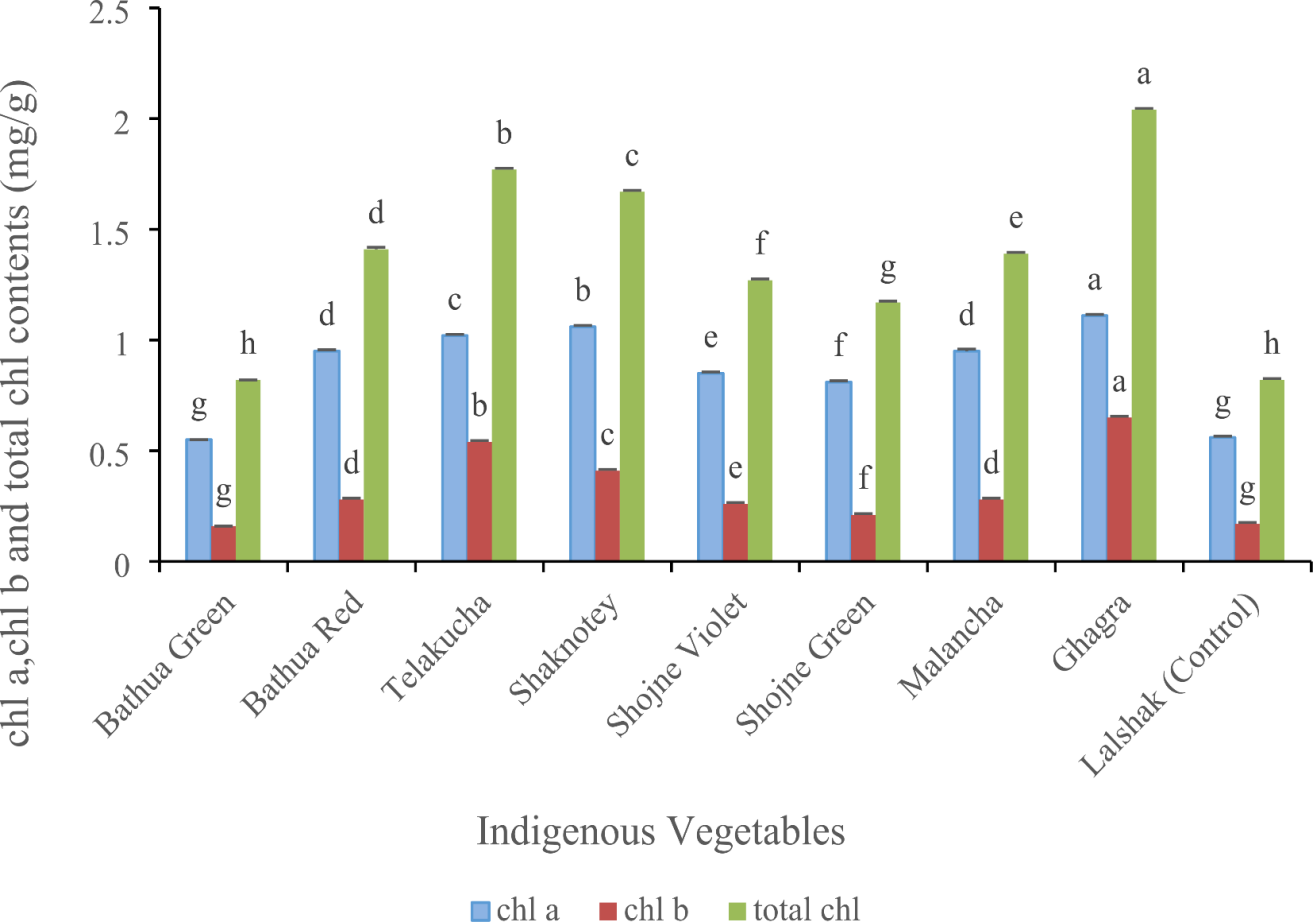
chl a, chl *b*, and total chl contents in different indigenous vegetables. [Error bars indicate standard error. Different letters on the bars show significant differences at < 0.05.]

There was a significant variation among the indigenous vegetables in respects of chlorophyll a, chlorophyll b and total chlorophyll contents (Fig. 2). Among the varieties, ghagra produced the highest chl a (1.11 mg/g), chl b (0.65 mg/g) and total chl contents (2.04 mg/g); whereas, shaknotey genotype had the second highest amount of chl a (1.06 mg/g) and chl b (0.41 mg/g). And the second highest total chl content found in telakucha (1.77 mg/g), while shaknotey had 1.67 mg/g total chl. Mean-while, the lowest value of chl a (0.55 mg/g) and chl b (0.16 mg/g) and total chl (0.82 mg/g) found in bathua green variety, which was statistically similar with the test vegetable -lalshak. The results showed that there was a significant variation among the indigenous vegetables (*p* < 0.05) on the content of chlorophyll. Though bathua red variety shows average concentration of total chl (1.41 mg/g), but it has lower rate of chl b (0.28 mg/g), whereas, shojne green (1.17 mg/g) and shojne violet (1.27 mg/g) had closer value of total chl with the lower rate of chl b (0.21 mg/g and 0.26 mg/g respectively). In our study, control crop lalshak showed lower rate of chlorophyll content (chl a 0.56 mg/g, chl b 0.17 mg/g and total chl 0.82 mg/g).

#### Total carotenoids and β-carotene (mg/100 g)

**Fig. 3:**
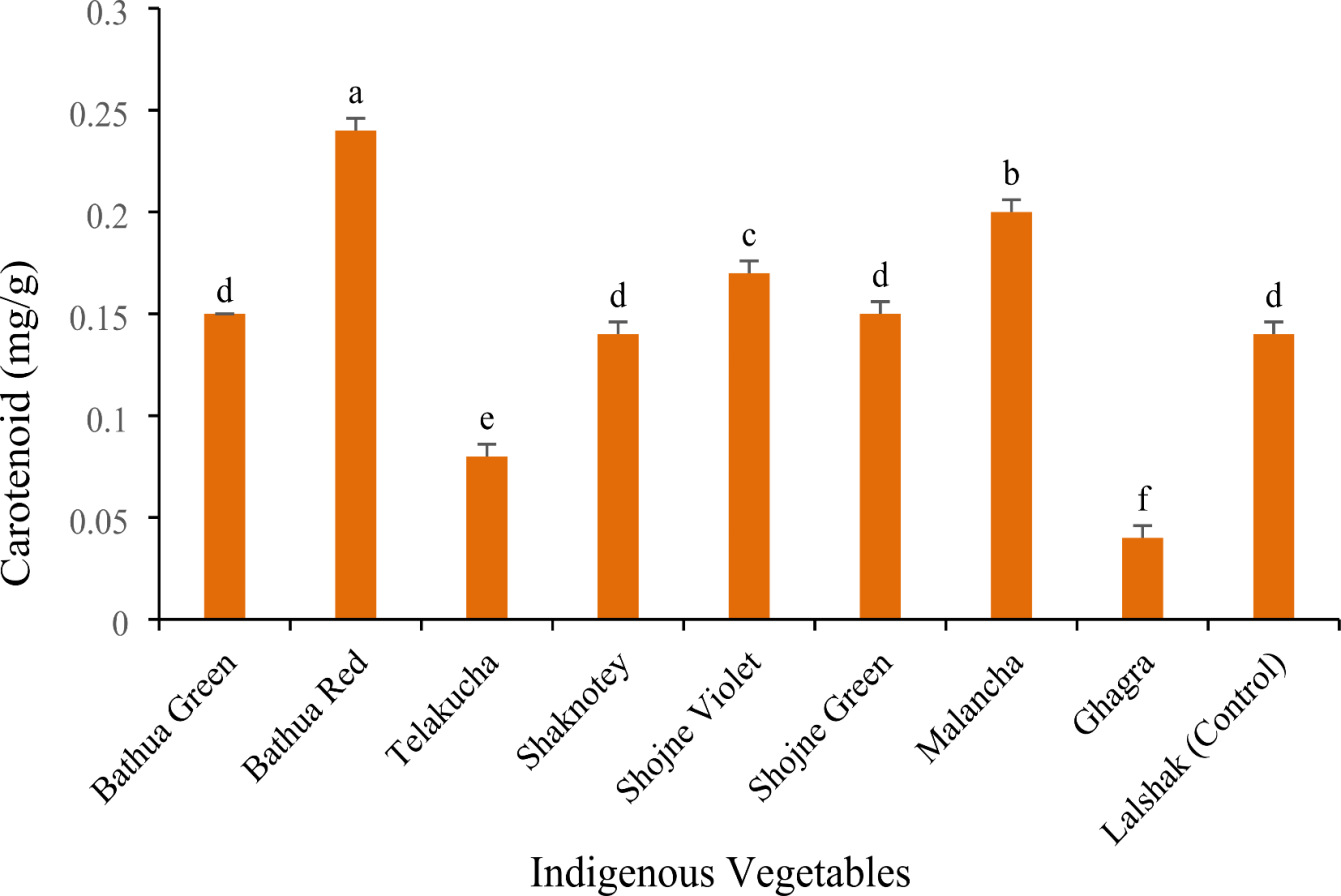
Carotenoid content in different indigenous vegetables. [Error bars indicate standard error. Different letters on the bars show significant differences at <0.05.]

Significant differences in carotenoid concentrations were found among the leaves of different indigenous vegetables (Fig. 3). Among these, the lowest concentration of total carotenoid was observed in ghagra (0.04 mg/g) and the highest (0.24 mg/g) in bathua red followed by malancha (0.2 mg/g) (Fig. 3). Moreover, bathua green and shaknotey contained the similar amount of carotenoid as that of lalshak (control). While this study showed, shojne violet and shojne green variety contained 0.17 mg/g, 0.15 mg/g carotenoids respectively, followed by control crop BARI lalshak-1 0.14 mg/g.

**Fig. 4:**
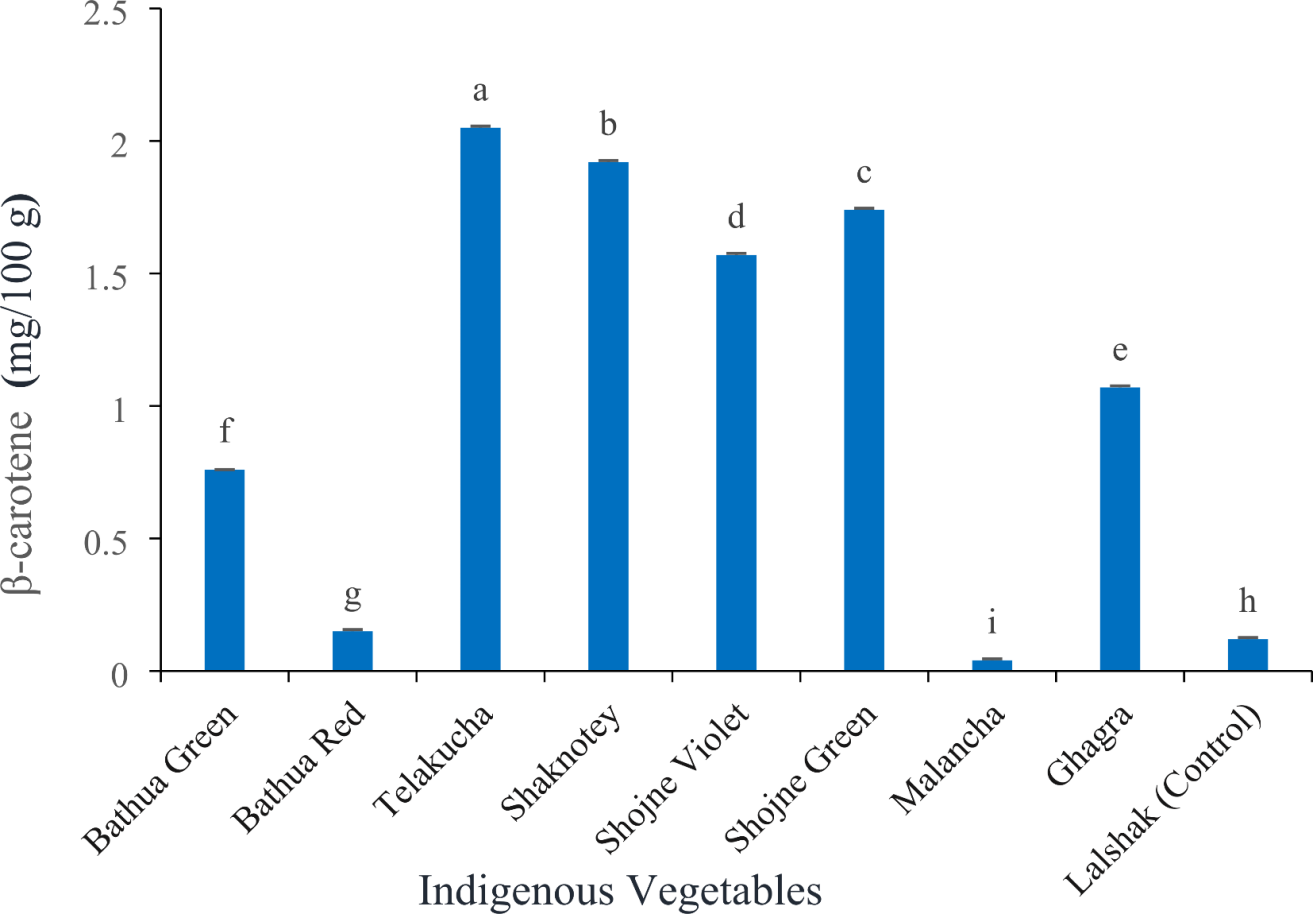
β-carotene content in different indigenous vegetables. [Error bars indicate standard error. Different letters on the bars show significant differences at < 0.05.]

There was also a significant (*p* < 0.05) variation observed in indigenous vegetables and control species on β-carotene content (Fig. 4). Telakucha showed the highest β-carotene (2.05 mg/100 g) while the lowest was in malancha (0.04 mg/100 g). In this study, the green vegetables like shaknotey (1.92 mg/100 g), shojne (1.57–1.74 mg/100 g), bathua green (0.76 mg/100 g), contained moderate amount of β-carotene. Bathua Red (0.15 mg/100 g) and control-lalshak (0.12 mg/100 g) also contained a lower amount of β-carotene.

#### Ascorbic acid content (vitamin-C) (mg/100 g)

**Fig. 5:**
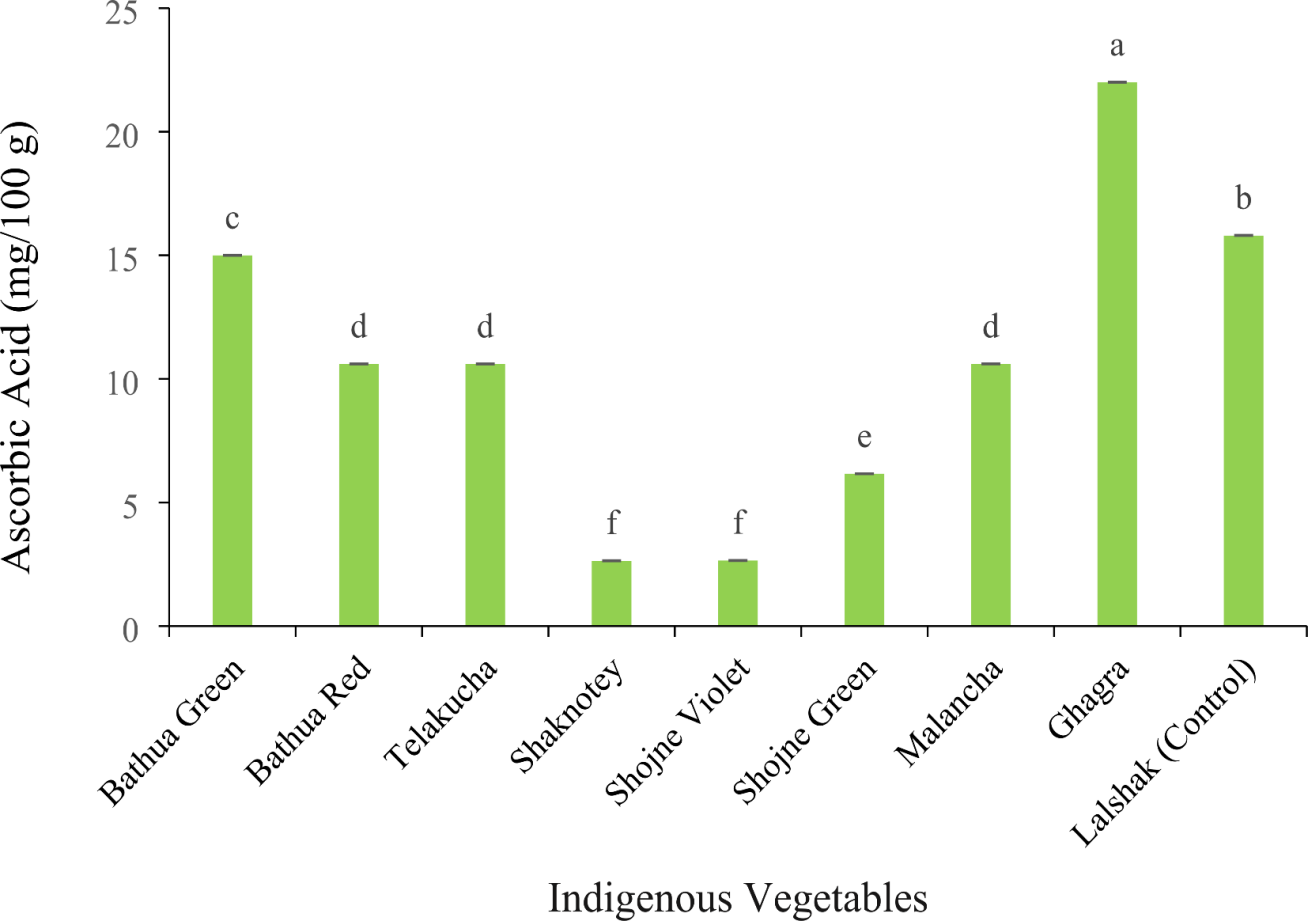
Ascorbic Acid content in different indigenous vegetables. [Error bars indicate standard error. Different letters on the bars show significant differences at < 0.05.]

A significant variation was observed among the studied indigenous vegetables considering ascorbic acid content (Fig. 5). As observed, ghagra contained the highest amount of ascorbic acid 22.0 (mg/100 g), while the control plant lalshak contained 15.8 mg/100 g, However, shaknotey and shojne violet had the lowest value of AA (ascorbic acid), which is statistically similar to each other (2.65 mg/100 g). Bathua red (10.6 mg/100 g), telakucha (10.6 mg/100 g) and malancha (10.6 mg/100 g) had the moderate amount of ascorbic acid and statistically identical with one another.

#### Anthocyanin content (µg/g)

**Fig. 6:**
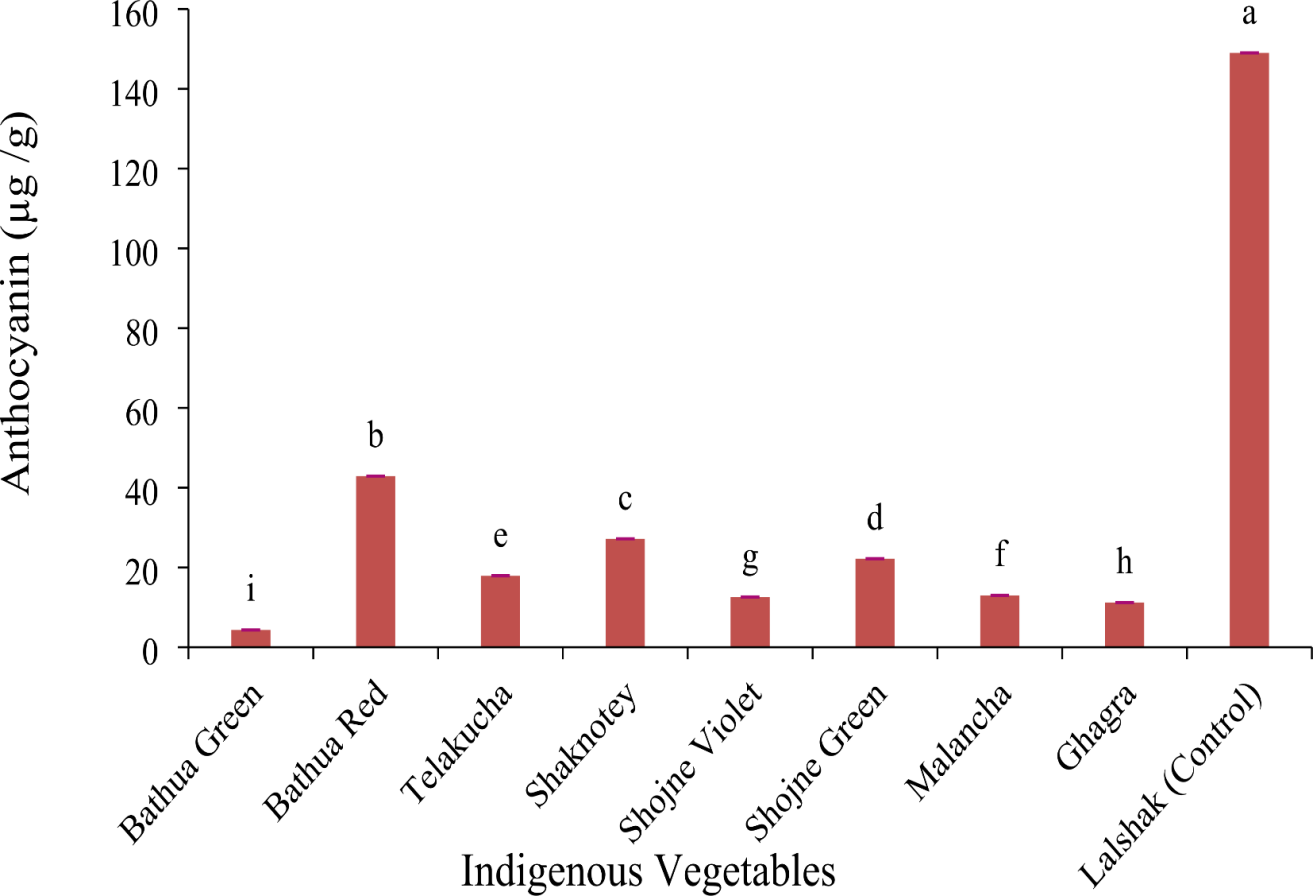
Anthocyanin content in different indigenous vegetables. [Error bars indicate standard error. Different letters on the bars show significant differences at < 0.05.]

There was a significant variation observed among the studied indigenous vegetables considering anthocyanin. The highest value of anthocyanin was found in lalshak (control) (149.0 µg/g) compared to the indigenous vegetables (Fig. 6). Bathua green had the lowest 4.34 µg/g anthocyanin content followed by ghagra 11.2 µg/g. Whereas, telakucha and shaknotey contained statistically closer amount of anthocyanin (17.9 µg/g). Among the other vegetables, bathua red shows higher 42.9 µg/g anthocyanin content (Fig. 5) than the same species of bathua green (4.34 µg/g).

#### Minerals (mg/g) and moisture content (%) of selected indigenous vegetables

Table 2 shows the mineral contents of selected indigenous leafy vegetables, which are principal sources of dietary minerals including iron, zinc, calcium, magnesium, sodium, phosphorus, and potassium. The concentration of calcium in the sample varied from 2.15 mg/g to 7.88 mg/g. Among these the highest concentration of Ca (7.88 mg/g) was found in telakucha and the lowest value of Ca (2.15 mg/g) in shojne violet, whereas the same species shojne green contained 5.16 mg/g. Notable amount of Ca also found in Bathua red (7.46 mg/g), malancha (7.43 mg/g) and shaknotey (7.37 mg/g) which are statistically significant (Table 2). The amounts of Mg were more or less close to each other ranged 0.79–0.89 mg/g, where malancha contained the highest amount of Mg (0.89 mg/g). Among the all parameters of evaluated minerals, K content (ranged 49.4–79.4 mg/g) was higher in selected indigenous vegetables (Table 2), where highest and lowest amount found in bathua green (79.4 mg/g) and malancha (49.4 mg/g), respectively. Ghagra and shojne green contained the highest amount of Na (1.94 mg/g) and Fe (1.63 mg/g), respectively. But the same species, shojne violet showed the lowest amount of Na (1.26 mg/g). The control BARI lalshak-1, ghagra and malancha showed the highest value of Na (mg/g) with 1.9, 1.94, 1.93 respectively, which were numerically similar to one another (Table 2).

**Table 2:** Mineral compositions of different indigenous vegetables.

Vegetables	Ca (mg/g)	Mg (mg/g)	Fe (mg/g)	Na (mg/g)	K (mg/g)
BathuaGreen	7.87 ± 0.012 b	0.84 ± 0.012 c	0.63 ± 0.012 h	1.61 ± 0.012 e	79.4 ± 0.006 a
BathuaRed	7.46 ± 0.012 d	0.84 ± 0.012 c	0.36 ± 0.012 i	1.87 ± 0.012 d	71.1 ± 0.006 c
Telakucha	7.88 ± 0.012 a	0.82 ± 0.012 e	0.9 ± 0.012 f	1.52 ± 0.012 f	68.8 ± 0.006 d
Shaknotey	7.37 ± 0.012 f	0.82 ± 0.012 e	1.23 ± 0.012 e	1.48 ± 0.012 h	67.2 ± 0.006 f
ShojneViolet	2.15 ± 0.012 i	0.79 ± 0.012 g	1.34 ± 0.012 c	1.26 ± 0.012 i	73.5 ± 0.006 b
ShojneGreen	5.16 ± 0.012 g	0.83 ± 0.012 d	1.63 ± 0.012 a	1.51 ± 0.012 g	68.0 ± 0.006 e
Malancha	7.43 ± 0.012 e	0.89 ± 0.012 a	1.3 ± 0.012 d	1.93 ± 0.012 b	49.4 ± 0.006 i
Ghagra	7.82 ± 0.012 c	0.80 ± 0.012 f	0.82 ± 0.012 g	1.94 ± 0.012 a	62.0 ± 0.006 h
Lalshak (Test)	3.59 ± 0.012 h	0.86 ± 0.012 b	1.4 ± 0.020 b	1.9 ± 0.012 c	65.6 ± 0.006 g

[Na = sodium; K = potassium; Ca = calcium; Mg = magnesium; Fe = iron. Data presented as means ± standard deviation in each column followed by different letters are significantly different at *p* < 0.05 as determined by Honestly significant difference test (Tukey’s HSD test) using the R program].

**Fig. 7:**
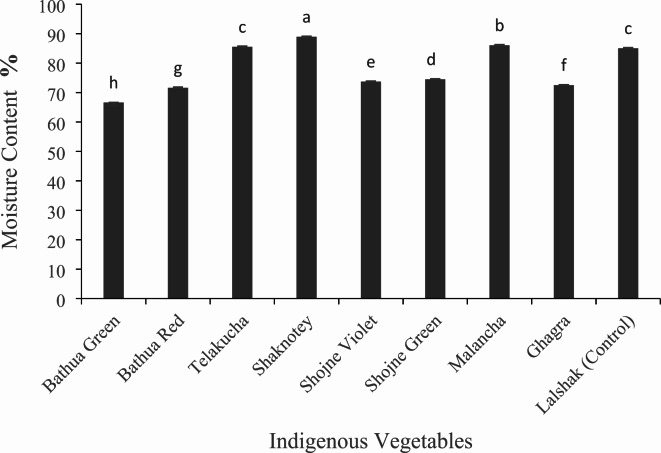
Moisture content in different indigenous vegetables. [Error bars indicate standard error. Different letters on the bars show significant differences at < 0.05.]

Meanwhile, the vegetable leaves exhibited a significant variation in moisture content (Fig. 7), with the greatest in shaknotey (88.97 %). This value was equivalent to that of malancha (86.07 %), folloed by telakucha (85.55 %) and the control BARI-lalshak 1 (85.07 %). While the water content in bathua green was significantly low, measuring at 66.62 % followed by ghagra (72.55 %). On the other hand, shojne green and shojne violet showed statistically significant value of 74.52 % and 73.73 % respectively.

### Bio-active compounds of indigenous vegetables

#### Total phenolic content (TPC) (mg GAE/100 g FW)

**Fig. 8:**
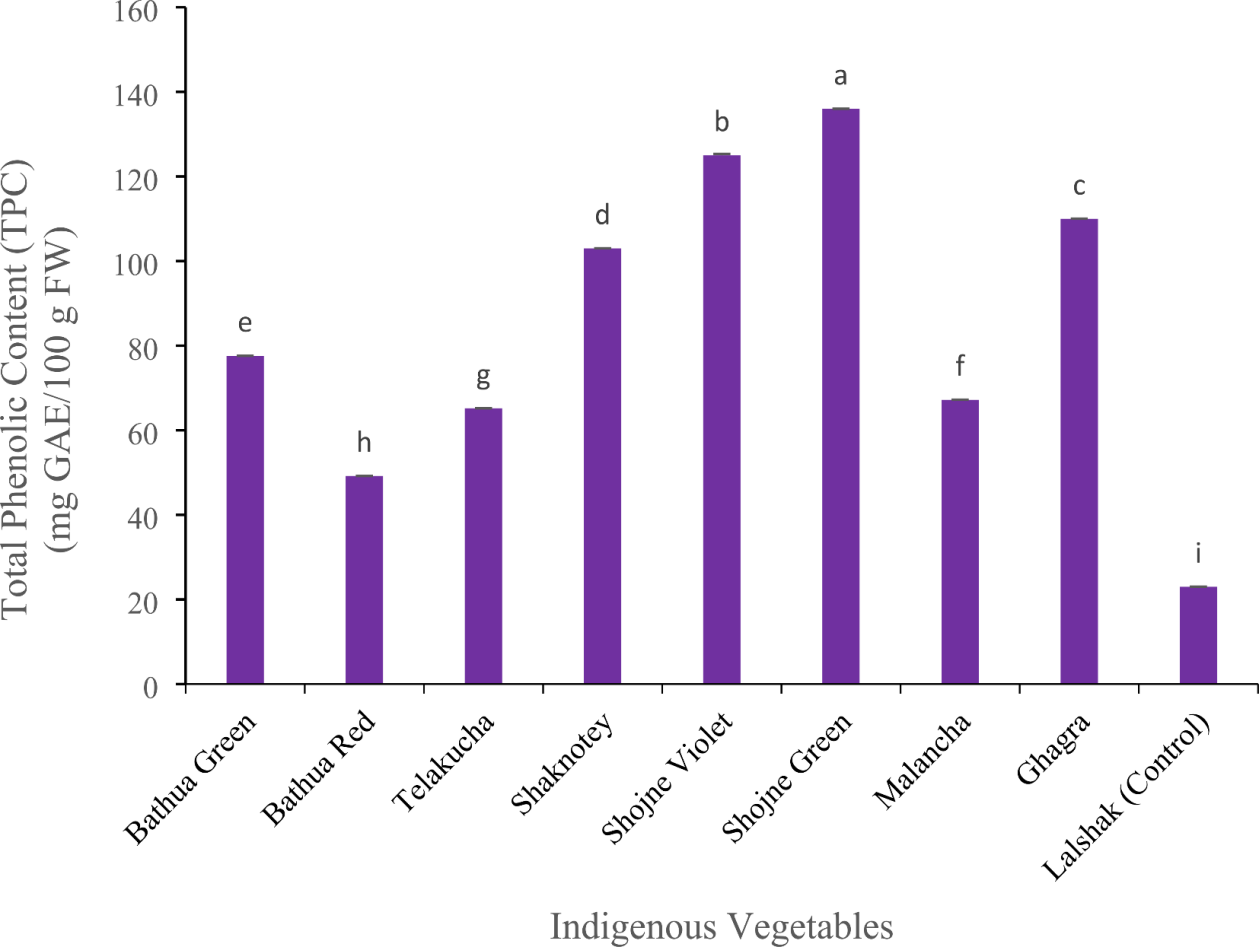
Total phenolic content in different indigenous vegetables. [Error bars indicate standard error. Different letters on the bars show significant differences at < 0.05.]

The valuable metabolite-phenolic content in different vegetables has been evaluated in this study and are displayed in Fig. 8. Among the vegetables, the two varieties of shojne have higher amount of phenolic content; where shojne green contain the highest 136.0 mg GAE/100 g FW, followed by shojne violet 125.0 mg GAE/100 g FW. The test vegetable BARI Lalshak-1 contain 23.0 mg GAE/100 g FW, which is much lower than the IVs. The other vegetables have been also found to have varying levels of phenols. The same species of bathua green and red variety have significant variation in total phenolic contents; 77.6 mg GAE/100 g in bathua green and 49.2 mg GAE/100 g in bathua red. However, shaknotey (103.0 mg GAE/100 g) and ghagra (110.0 mg GAE/100 g) have high amount of TPC. On the other hand, telakucha and malancha shows quite similar amount of TPC, ranges from 65.2 to 67.2 mg GAE/100 g.

#### Total flavonoid content (TFC) (mg QE/100 g FW)

**Fig. 9:**
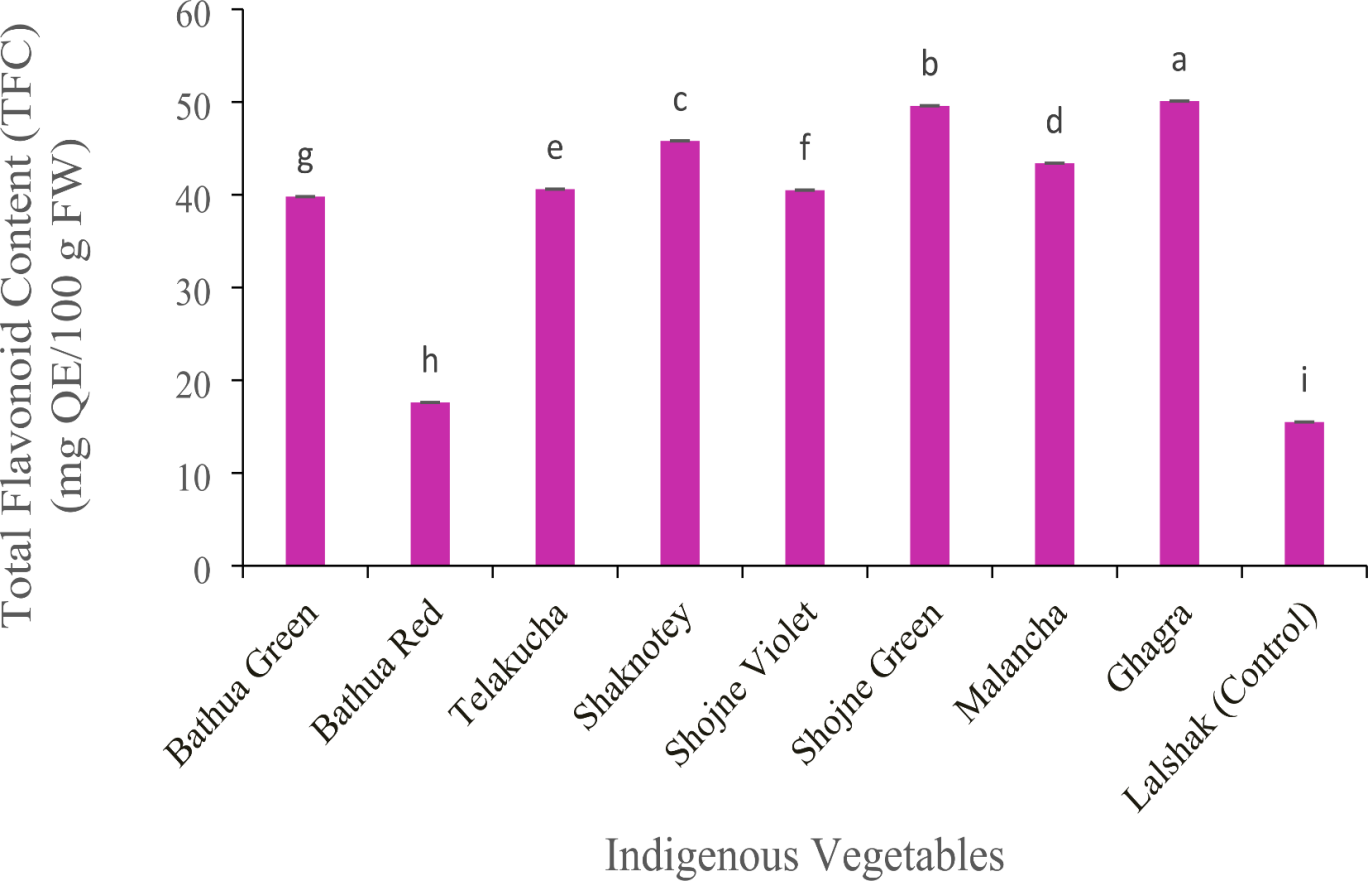
Total flavonoid content in different indigenous vegetables. [Error bars indicate standard error. Different letters on the bars show significant differences at < 0.05.]

There was a significant difference observed among the indigenous vegetables regarding flavonoid content (Fig. 9). The highest value of total flavonoid content was found 50.1 mg QE/100 g FW in ghagra. Result of our study showed higher TFC 49.6 QE/100 g FW in shojne green, which is almost similar with shaknotey (45.8 QE/100 g FW) and the lowest 15.5 mg QE/100 g FW observed in control vegetable BARI–lalshak 1 and bathua red 17.6 mg QE/100 g FW followed by bathua green (39.8 mg QE/100 g FW) and other vegetables.

#### Total antioxidant activity (TAA) (µg/ml)

**Fig. 10:**
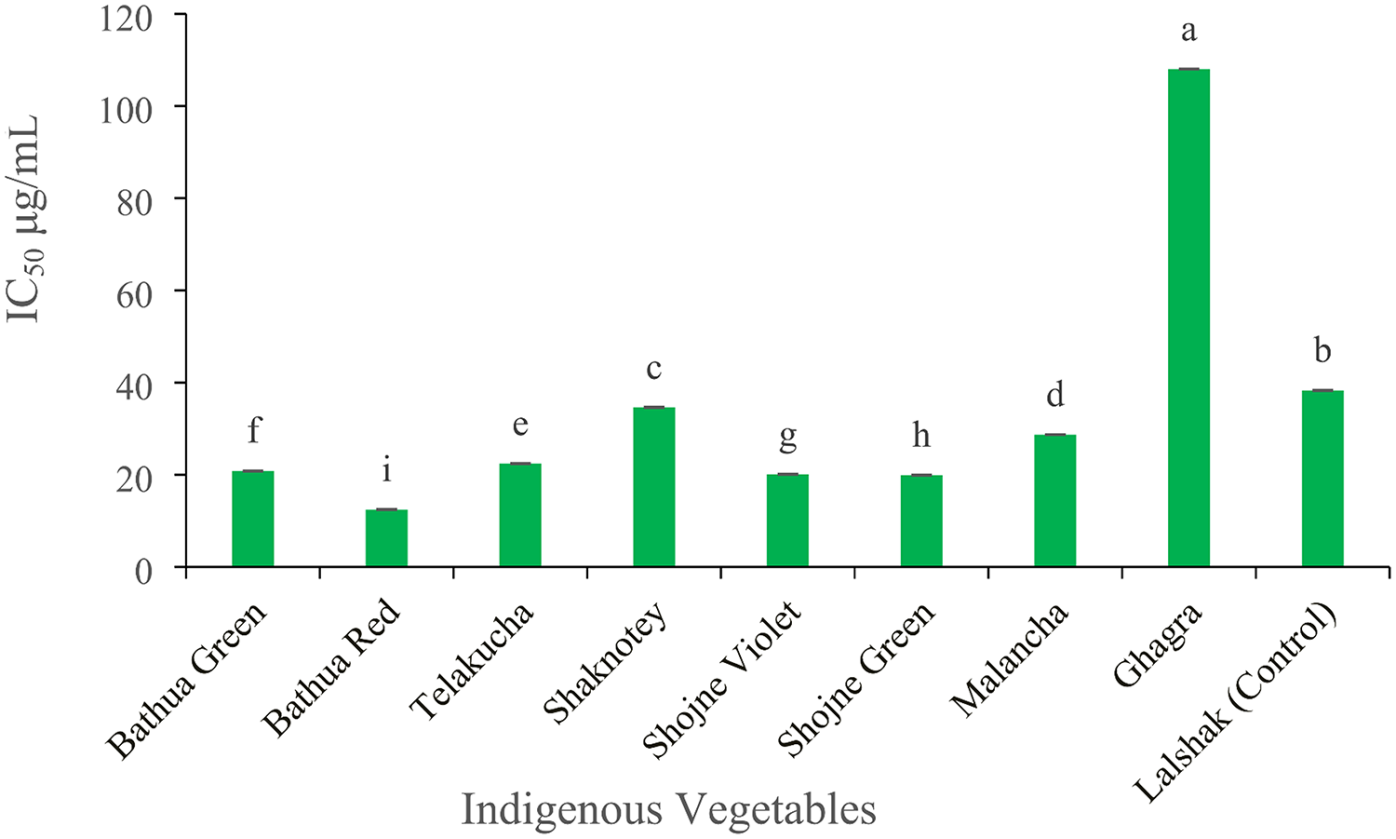
Antioxidant content in different indigenous vegetables. [Error bars indicate standard error. Different letters on the bars show significant differences at < 0.05.]

Figure 10 shows that the IC_50_ values of selected indigenous vegetables had a substantial impact on their ability to scavenge free radicals (*p* < 0.05). Among the vegetables, ghagra denoted the highest (108.0 µg/mL) IC_50_ values, followed by control plant lalshak with the IC_50_ value 38.3 µg/mL. However, the lowest 12.4 µg/mL of IC_50_ value showed in bathua red. And the other selected IVs showed more or less close to IC_50_ value in Fig. 10.

As the antioxidant activity of the indigenous vegetables were assessed by determining the IC_50_ value, which represents the concentration of the sample needed to block 50% of DPPH free radicals. Therefore, in the DPPH experiment, greater IC_50_ values indicate lesser antioxidant activity, and vice versa. Thus, bathua red had the highest antioxidant activity and ghagra had the lowest potentiality among the all studied vegetables.

### Correlation coefficient analysis

**Fig. 11:**
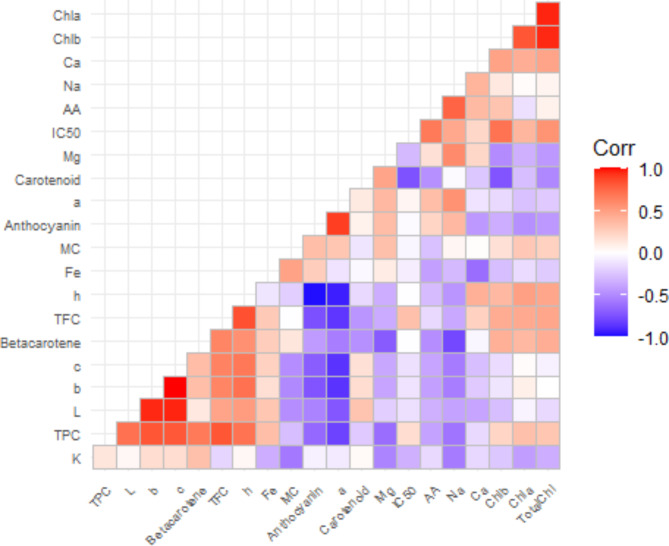
Correlation coefficient for different bio-chemical and mineral composition in the different indigenous vegetables.

Pearson’s correlation was assessed to evaluate the interrelationships among the studied variables. Correlation matrix revealed both strong positive and negative correlation among the variables (Fig. 11). If one variable response increased, then the other one would be increased too for positive correlation and vice versa for negative correlation. The Fig. 11 was distributed from positive to negative values indicated by the red to purple-colored squares. White color cells were considered as non-significant (zero) relationship among the variables at 5% level of significance. Red color square (0.5–1.0) denotes the stronger correlation between the two respective variables. From the total variables, there was a strong negative correlation (R^2^ = − 0.13, − 0.28, − 0.34, − 0.15, − 0.4, − 0.48) between the chlorophyll pigments and vitamin C, carotenoid, Mg, Fe, K, and anthocyanin. In addition, there was also a significant negative association between the chlorophyll pigments and L* (R^2^ = − 0.16), a* (R^2^ = − 0.23) respectively, which means that increase in chlorophyll pigments brings about decrease in these parameter’s value. Highly significant positive correlation chl a and chl b were exhibited among pigments and with total chl (R^2^ = 0.96; 0.95), while chl b had strong negative correlation with carotenoid (R^2^ = − 0.74). The total carotenoid concentration exhibited a significant positive correlation with L (R^2^ = 0.31), additionally negative link with h° and moisture content % (R^2^ =− 0.16, − 0.11). β-carotene had significant correlation coefficients with TPC, TFC and hue angle R^2^ = 0.67, 0.62 and 0.57 respectively, while it showed insignificant (no relationship) associations with antioxidant (R^2^ = − 0.01) and in addition negative association with a* (R^2^ = − 0.56). Considering, the antioxidant features IC_50_ had positive correlation with chl b (R^2^ = 0.70), total chl (R^2^ = 0.55) and AA (ascorbic acid) (R^2^ = 0.67) content, whereas it exhibited a substantial negative association with Fe, L* and b* and c* (R^2^ = − 0.07, − 0.13, − 0.12 and −  0.13). Among the mineral composition Mg had strongly negative correlation with β-carotene, b*and c* (R^2^ = − 0.71, − 0.39, − 0.38), while it had positive correlation with Na and MC% (R^2^ = 0.59, 0.33). Considering the anthocyanin variable, it had strongly negative correlation with TPC (R^2^ = − 0.64), TFC (R^2^ = − 0.76), as well as with b*, c* and h° (R^2^ = − 0.65, − 0.74, − 0.70 and − 0.98). Furthermore, it was also demonstrated a significant positive relationship with a* (R^2^ = 0.90).

### Principal component analysis (PCA)

Principal component analysis simplified the complex data by transforming the number of correlated variables into a smaller number of variables to figure out the most significant traits. The relationships (similarities and dissimilarities) among different indices were graphically displayed in a biplot (Fig. 13) of Dim 1 (PC1) and Dim 2 (PC2). The first two principal components (PC) contributed enough to explain the maximum (about 62.4 %) of the pattern variations. Individually, only PC1 and PC2 could explain 38.1 % and 24.3 % of the total data variance which were shown in Fig. 13. The positive results showed along with Dim1 were characterized for AA (ascorbic acid), carotenoid, mineral composition (Mg and Na), anthocyanin, distinct color directions a* and MC ((ranging from 0.07 to 0.33), while the negative scores along with Dim1 corresponded to chlorophyll pigments (chl a, chl b and total chl), beta-carotene, TPC, TFC, IC_50_, mineral composition (Ca, Fe and K), lightness L*, distinct color directions b* and chroma of the color c*(ranging from − 0.02 to − 0.33).

**Fig. 12:**
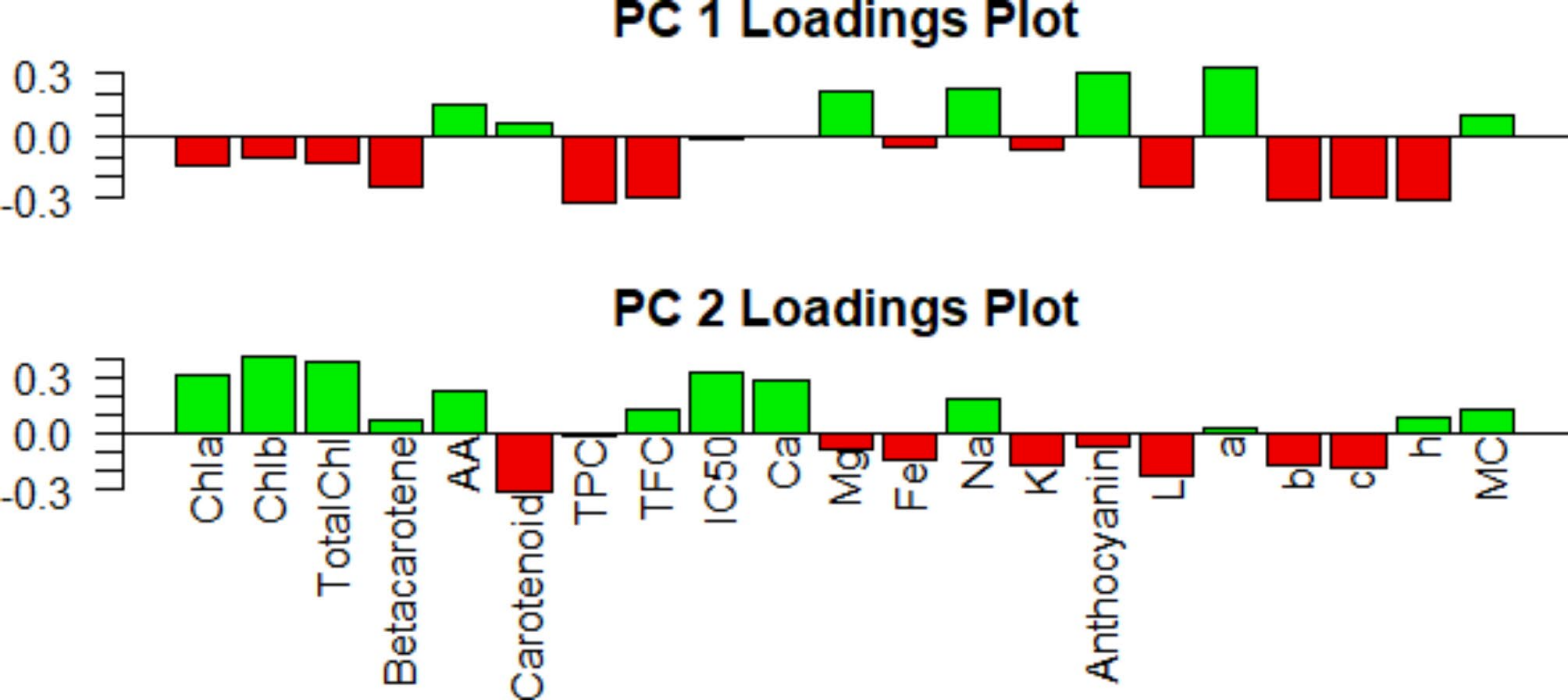
Loadings plot for PC1 and PC2 of leaves color, nutritional and antioxidant characteristics of different indigenous vegetables.

Conversely, nine variables, namely carotenoid, TPC, Mg, Fe, K, anthocyanin, L*, b* and c* exhibited a negative score along with Dim2. On the contrary, the positive scores along with Dim2 characterized for chlorophyll pigments (chl a, chl b and total chl), beta-carotene, ascorbic acid (AA), TFC, IC_50_, Ca, Na, a*, h° and and MC (Fig. 12). Considering in both PC 1 and PC 2 loading plot ascorbic acid, Na, a* and MC variables are positively contributed and Fe, K, L*, b* and c* are negatively contribute here.

**Fig. 13:**
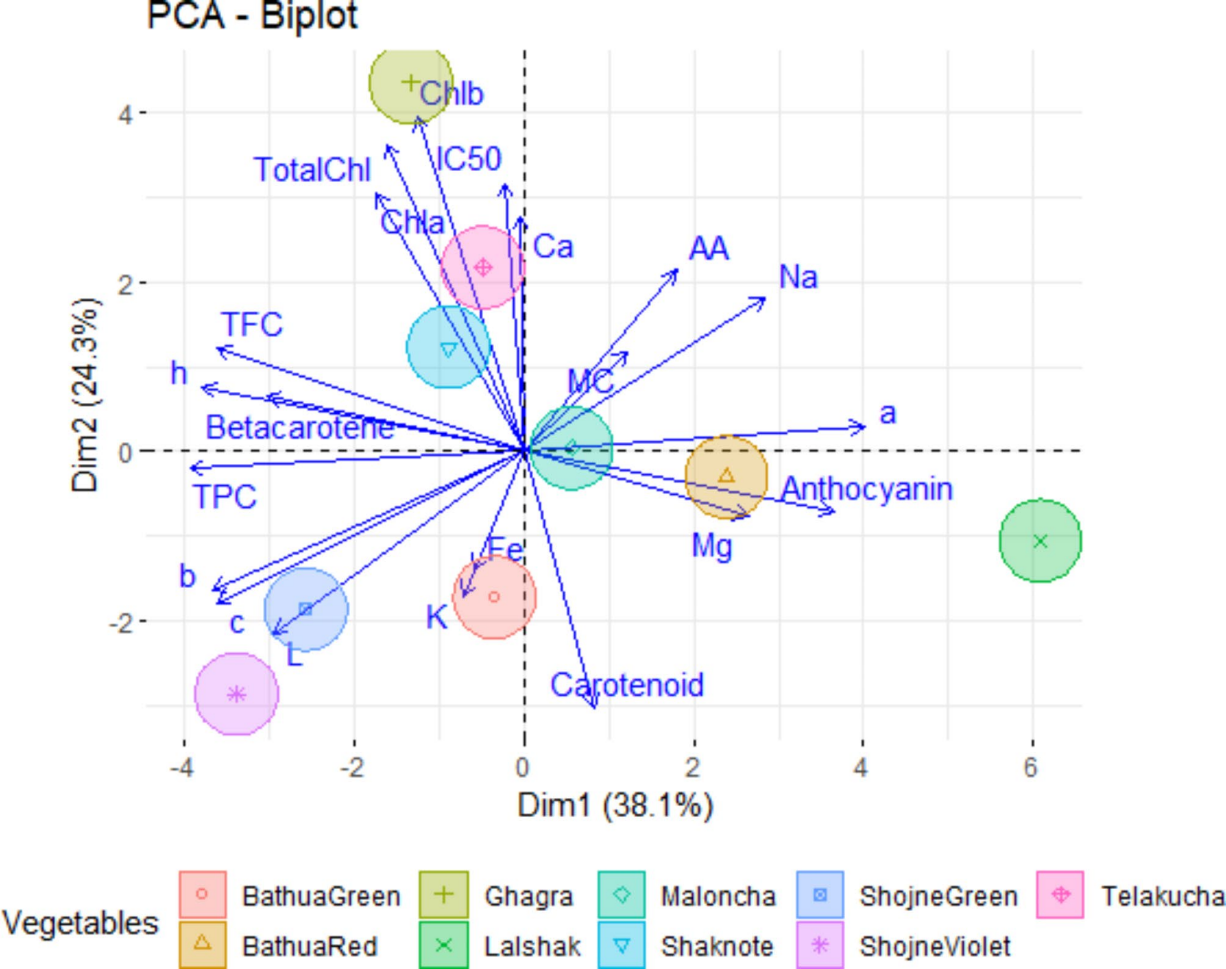
Biplot diagram of principal component analysis of different indigenous vegetables based on their nutritional attribute.

A hierarchic PCA-biplot analyses were performed integrating all the nutritional information for the eight vegetables, where four vegetables (bathua red, malancha and lalshak-control) are positively and other six vegetables (bathua green, shojne green, shojne violet, telakucha, shaknotey and ghagra) are negatively represent in Dim1, while in Dim 2 the three vegetables (shaknotey, telakucha and ghagra) are positive and others are negative. Results from the biplot of the vegetable characteristics revealed that malancha and the variable MC, AA, Na and a* located in a distinct position in relation to the Dim1 and Dim2, where most of the variables were showing a positive correlation to malancha. There is no contribution of Ca as we can see in Fig. 13. However, the biplot displays both the observations and variables in a given orientation along the PC axis (Dim1 and Dim2) concurrently. The orientation of the variable arrows signifies the direction in which the contribution of the related variable experiences the greatest rise, while the length of the arrows represents the magnitude of the change in that direction. Overall, the clustering and PCA-biplot analyses permitted the identification of theoretical vegetable combinations to develop well-balanced nutritional and bio-active properties.

### Cluster dendrogram

**Fig. 14:**
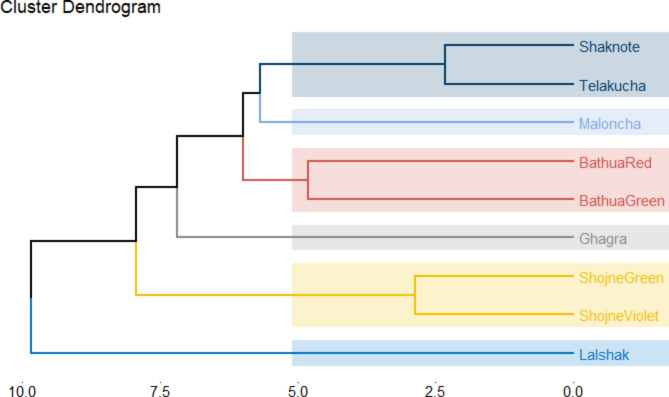
Cluster dendrogram of different indigenous vegetable.

Cluster analysis was conducted using the K-means algorithm to group eight indigenous vegetables and one test crop based on some quantitative attributes. To do this, a dendrogram was constructed and then divided at a rescaled distance of 10.0. This cluster analysis of the dendrogram showed that the studied vegetables were divided into some most significant clusters with remarkable contributions in the quality evaluation. Based on the degree of divergence vegetables were grouped into four main clusters (Fig. 14), in which the control crop BARI-lalshak 1 is completely different and distinctly in isolated cluster (I). The second cluster was further divided into one sub clusters including shojne green and shojne violet. Cluster (III) represent ghagra in isolated zone, which is also different from other IV’s. The forth cluster including with another 5 vagetables and they are bathua red and green varieties, malancha, telakucha and shaknotey. In where malancha itself make a small cluster and bathua species -green and red are relevant to each other, while, telakucha and shaknotey make another small cluster. Finally comparing the indigenous vegetables, ghagra and malancha are completely different and containing the substantial length of the total cluster.

## Discussion

Vegetables are an important part of the human diet and a major source of biologically active substances such as vitamins and secondary metabolites^35^. They are valued mainly for their valuable food ingredients- vitamin and mineral contents which can be successfully utilized to build up and repair the human body^36^. Indigenous vegetables play a crucial role in sustaining local economies, human nutrition, health, and social systems. Bangladesh, being mostly an agricultural country, has a diverse range of ecological conditions that support the growth of various crops and vegetables in different regions. In isolated areas, such as villages and tribal communities, many people consume various indigenous vegetables that are not commonly found in mainstream agriculture. Leaves are the most commonly utilized plant components among culinary plants. They believe that it contains nutritional content that is important for their food and nutritional security. The characteristics of vegetables are connected to the individuals who consume them regularly.

In the investigation, it was found that the leaves of indigenous vegetable with the highest L* value was shojne violet, which had a light (pale green) color. Additionally, it had also the highest b* value. The red color leaves with the maximum a* value was BARI-lalshak 1. These findings were congruent with the visually seen colors of the leaves of the selected indigenous vegetables (Table 1). Prior studies have indicated that anthocyanins contribute to the development of red to purple hues^23^. The results of this investigation have also demonstrated a resemblance to the previous study, indicating that BARI-lalshak 1 had highest value of anthocyanin. It has been also considered that red color genotype had high redness and yellowness values indicating the presence of abundant pigments of anthocyanins^17^. The correlation study has also demonstrated a strong positive association between the total anthocyanin and a* value, as indicated by the multivariate analyses as well and at the same time chlorophyll pigments showed a negative association with a*. In contrast, the green color genotype showed high greenness and blueness indicating the presence of minimum pigments of anthocyanins. However, BARI-lalshak 1 (red color), have demonstrated a high capacity for accumulating anthocyanin and lowest amount of chlorophyll, contribute to the yellow to red colors observed in the plants. Additionally, Shojne varieties showed lowest value of a*and poor amount of total chlorophyll that represent its pale green color leaves. As, lalshak, bathua red has redness in their leaves and bathua green, sojne, ghagra has dark green color leaves, they show high and low concentrations of anthocyanin respectively. Anthocyanin act as antioxidants as well as anti-cancerous agents^37^. Result of this study showed the highest value of anthocyanin in control vegetable BARI-lalshak 1 (149.0 µg/g) compared to the indigenous vegetables (Fig. 6). In contrast, the green color genotype showed high greenness and blueness indicating the presence of minimum pigments of anthocyanins. Among the other vegetables, bathua red shows higher 42.9 µg/g anthocyanin content (Fig. 6), which may be because of their red and green color variation.

This study examined the link between the overall antioxidant activity (measured by the IC_50_ value) of several indigenous vegetables and their secondary metabolites. The results showed a positive correlation between the IC_50_ value and the presence of chlorophyll pigments, ascorbic acid, phenolic and flavonoid content and also calcium, sodium. In addition, there is a clear negative association between beta-carotene, carotenoid and the levels of magnesium (Mg), potassium (K) and iron (Fe), as indicated by the IC_50_ value. Result shows that the IC_50_ values of selected indigenous vegetables had a substantial impact on their ability to scavenge free radicals (*p* < 0.05). As the antioxidant activity of the indigenous vegetables were assessed by determining the IC_50_ value, which represents the concentration of the sample needed to block 50% of DPPH free radicals. Therefore, in the DPPH experiment, greater IC_50_ values indicate lesser antioxidant activity, and vice versa. Thus, bathua red had the highest antioxidant activity and ghagra had the lowest potentiality among the all studied vegetables. Leaves color is a key indicator of the leafy vegetables for evaluating the antioxidant potentiality^17^. It has been revealed that all antioxidant constituents of dark green color leave of ghagra had strong antioxidant activity in this present study. But in another study, the IC_50_ values of the methanolic extract of ghagra were 0.02 mg/mL and 0.09 mg/mL had significant antioxidant activity^38^. Due to the lower the IC_50_ value, the more potent is the substance at scavenging DPPH and this implies a higher antioxidant activity^39^ and this kind of variation might be happening for collecting the vegetables from different locality with different growing factors. Also, carotenoids have strong antioxidant capacity. In this study bathua red contains high concentrations of carotenoids (Fig. 3) and also lower rate of phenolic compounds (Fig. 8), and thus it showed higher antioxidant activity than other vegetables.

Mineral substances also contribute to the development of leaves color by combining with flavonoids to create supramolecular pigments known as metal complexes. Result showed that selected indigenous vegetables contained some amounts of several micronutrients because of containing antioxidants and phytochemicals in rich amounts such as calcium, magnesium, sodium, potassium, iron, which were lower than the literature. According to the literature, compared to other food sources indigenous vegetables contain more micronutrients and various amounts of compounds that are needed to address nutritional and health requirements^40^. As nutrients and minerals generally comes from the soil (such as K and P moves from the soil into plant roots by diffusion), growing region, planting material, soil condition and environmental factors might be responsible for this kind of variation among the indigenous vegetables. Our investigation has revealed a weak to negative association between the total flavonoid levels and mineral matters, anthocyanin, a*, and chlorophyll, by which the selected indigenous vegetables showed their green color variation (light green to dark green).

Flavonoids are an important class of natural products; particularly, they belong to a class of plant secondary metabolites having a polyphenolic structure, widely found in several parts of the vegetables. As most phenolic compounds and flavonoids were found in the leaf extract due to photosynthesis in leaves, flavonoid biosynthetic pathway precursors (Shikimic acid) are more abundant in leaves of vegetable^41^. Phenolics are the largest group of phytochemicals that account for most of the antioxidant activity in plants or plant products^42^. Flavonoid contents were reported in a variety of medicinal plants also. As Ghagra used as a traditional herbal medicine and has dark green leaves, it contains a good quantity of flavonoids contents that found in this study, which show also high anti-oxidant activity. Result of our study showed higher TFC 49.6 QE/100 g FW in shojne green and the lowest 15.5 mg QE/100 g FW observed in control vegetable –lalshak and bathua red 17.6 QE/100 g FW followed by other vegetables, which is compatible with previous study^43^, in where 16.75 mg QE/g TFC had been found in BARI–lalshak 1. On the contrary, the two varieties of shojne have higher amount of phenolic content and according to literature, 129.44mgGAE/g phenolic content were found in shojne^44^, which is compatible with the present study. The test vegetable BARI–lalshak 1 contain (23.0 mg GAE/100 g FW), which is much lower than the IVs. Because of control plant is red in color and the phenol content was ranked first in green leafy vegetables (GLVs) assayed when compared to other vegetables^45^. The other vegetables have been also found to have varying levels of phenols. As we can observe here, the same species of bathua green and red variety have significant variation in total phenolic contents. From this study, it can be said that the TPC of vegetables varies widely depending on the variety of vegetables. Comparison is difficult, as method of extraction solvents used; method adopted to extract phenol and standard compounds adopted varied among the studies vegetables.

In addition, the highest amount of chlorophyll was determined in ghagra, which was because of the upper part of a leaf is darker, owing to the high concentrations of chloroplasts present, as compared to the lighter bottom part^46^. On the other hand, the leaf texture is also contributing in the variation of the chlorophyll contents in plants. Some plants have thin leaves, but some have very thick leaves. Thicker leaves are either juicy (also called succulents) as in a cactus, or non-juicy (also called dicotyledons) as in Eucalyptus leaves. The leaves of dicotyledons are thick and non-juicy, as well as darker green, because they have dense chloroplasts that highly absorb sunlight, as similar with ghagra leaves. Moreover, succulent leaves tend to be lighter, as their cells are quite watery, so the concentration of chlorophyll on the surface is lower^46^. As, Shojne, shaknotey and telakucha have succulent leaves, so their cholorophyll contents were comparatively lower than ghagra as they had stippling leaves.

Furthermore, Rajyalakshmi et al.^47^ analyzed green leafy vegetables (GLV) where thirty-six were found to contain high total carotenoids (TC) contents ranging from 12.22 to 36.13%, whereas, the highest contents of carotenoid (38.765 mg/100 g) were recorded in the leaves of shojne. In addition, due to the red color, BARI lalshak-1 leaves had carotenoids^48^. It was claimed that red color genotypes are an excellent source of carotenoids (55.55 mg 100/g FW)^17^. But in this study, BARI lalshak-1 contained 0.14 mg/g carotenoid. But bathua red had the highest value in followed by malancha and the lowest concentration of total carotenoids was observed in ghagra. In addition, β-carotene in leafy vegetables was also reported higher previously (ranged from 36– 44 mg/100 g of dry wt)^49^. In this study, the green vegetables like shaknotey (1.92 mg/100 g), shojne (1.57–1.74 mg/100 g), bathua green (0.76 mg/100 g), contained a poor amount of β-carotene content. According to the literature, ascorbic acid content is well-established in indigenous vegetables and different experiments revealed the presence of high amounts of vitamin C. Arasaretnam et al.^50^ suggested that the green leafy vegetables contain considerable amounts of essential micronutrients in addition to the presence of high amounts of vitamin C ranged from 12.43 to 134.50 mg/100 g. As ghagra has very dark green color leaves, it has higher concentration of ascorbic acid (vit C). Previous reports claimed that ascorbic acid content of bathua fresh leaves was 220.97 to 377.65 mg^51^, which is much higher than this study. Because there are different varieties of the same species bathua are available, as here we used batua red and green variety hence, genotypic variations, growing region also responsible for the variation of ascorbic acid content in the bathua species. In some previous reports, shaknotey contained 21.41 mg/100 g ascorbic acid^52^ and lalshak contained 122.43 mg/100 g FW^17^, which were higher than the present study. This might be happened due to the variation of vegetable species, their physiological appearance (color) or growth habit and environmental factor. These might have happened because of using different species from different environmental locations; such as due to the age, cultivar, the climatic condition, their physiological appearance (color) or growth habit of the plant growing locations, assay techniques, extraction solutions, and chemicals as well.

The vegetables exhibit a high correlation with each other in terms of their nutritional content. We have undertaken PCA (Principal Component Analysis) based on linked variables to determine the most promising indigenous crops. The cluster-I comprises with the control crop BARI-lalshak 1 is completely different and distinctly in isolated. The second cluster was further divided into one sub clusters including shojne green and shojne violet, which is characterized by high levels of lightness, distinct color and chroma of the leaves color. Cluster (III) represent ghagra in isolated zone, which is different from other IV’s, mostly due to their high levels of antioxidant and ascorbic acid content. The fourth cluster including with another 5 vagetables and they are bathua red and green varieties, malancha, telakucha and shaknotey. In where malancha itself make a small cluster and rich in Mg content. Bathua green and red species are relevant to each other, while, telakucha and shaknotey make another small cluster with small amount of nutritional and bioactive compounds. Out of the 8 vegetables investigated, ghagra, shojne and bathua species stands out for their unique nutritional properties that differentiate these from the others and shows potential traits for future crops. These indigenous vegetables play a vital role in fulfilling the demand for nutritional, minerals and antioxidant compounds in the diet that is resemblance to the findings^53^. Along with commercially cultivated vegetables, these vegetables are considered to be a potential source of essential nutrients such as vitamins, minerals, proteins, fibers^54^ and are also good dietary sources of phytochemical compounds such as phenolic compounds, flavonoids, antioxidants and anthocyanin constituent activity^55^. The current data is crucial as a preliminary effort to present information on the nutritional and antioxidant properties of the examined IVs. However, it is advisable to conduct further research to clarify the specific elements of pigmentation, secondary metabolites, and antinutritional properties of the IVs using advanced techniques such as high-performance liquid chromatography (HPLC) or gas chromatography-mass spectrometry (GC-MS) in the future.

## Conclusion

The results demonstrate substantial diversity among the chosen native vegetables in regards to their antioxidant qualities, as well as the measured chemicals (total carotenoid, β-carotene content, anthocyanin, phenolic contents, flavonoid contents). The shojne violet color exhibits the highest values of lightness (L*), directions (b*), and chroma (c*), while the highest luminosity (h°) is exhibited in telakucha. Among the three, Shaknotey had the highest moisture level, whereas bathua green had a substantially lower moisture content, followed by ghagra. Ghagra displayed the most elevated concentrations of chlorophyll pigments, ascorbic acid, and flavonoid content. The indigenous vegetables, bathua red and telakucha, were discovered to possess the greatest levels of total carotenoids and β-carotene. The red variety of bathua had the second-highest concentration of anthocyanin and demonstrated the most effective antioxidant activity, as indicated by its IC_50_ value. On the other hand, the green variety of shojne had the highest phenolic content. The indigenous plants were rich in essential minerals like sodium, potassium, calcium, magnesium, and iron. Among these minerals, potassium had the highest concentration. The experiment clearly shown that indigenous plants possess a wealth of nutrients and bio-active compounds. Ghagra, shojne and bathua are more nutritious than the commonly consumed vegetable of BARI-lalshak 1. The results of principal component analysis (PCA) revealed a strong correlation between chlorophyll pigments, carotenoid, β-carotene content, h°, c*, a*, L*, TPC, TFC, Anthocyanin, and MC. These factors influenced the grouping of the examined indigenous vegetables into four clusters. Notably, ghagra, shojne, and bathua species were identified as superior plants, as evidenced by their distinct position compared to the other investigated indigenous vegetables. This finding indicates that ghagra, shojne and bathua have the capacity to become a locally grown, nutrient-dense crop in Bangladesh in the near future.

## Electronic supplementary material

Below is the link to the electronic supplementary material.

Supplementary Information 1.

## Data Availability

Data is provided within the manuscript or supplementary information files.

## Declarations

### Clinical trial number

This article does not involve any research regarding the clinical trial in human being or animals therefore there is no clinical trial number has been included in this manuscript.

## References

[1] FAO, IFAD, UNICEF, WFP and WHO. The State of Food Security and Nutrition in the World 2021. Transforming food systems for food security, improved nutrition and affordable healthy diets for all. Rome, FAO. 10.4060/cb4474en (2021).

[2] Li, X. & Siddique, K. H. M. Future smart food: harnessing the potential of neglected and underutilized species for Zero Hunger. *Matern Child. Nutr.* **16**(S3), e13008. 10.1111/mcn.13008 (2020). 33347726 10.1111/mcn.13008 PMC7752121

[3] Zafar, T., Mehra, A., Das, P., Shaik, B. & Malik, A. A. Demystifying the advanced interventions of genetics and modern breeding approaches for nutritional security and sustainability of neglected and underused crop species (NUCS). *Genet. Resour. Crop Evol.* **71**(2), 559–577 (2024).

[4] De la Penna, R. C., Ebert, A. W., Gniffke, P., Hanson, P. & Symonds, R. C. Genetic adjustment to changing climates: vegetables. In *Crop Adaptation to Climate Change* (eds. Yadav, S.S. et al.) 396–410 (Wiley, 2011).

[5] Hughes, J. D. A. & Ebert, A. W. Research and development of underutilized plant species: the role of vegetables in assuring food and nutritional security. In *II International Symposium on Underutilized Plant Species: Crops for the Future-Beyond Food Security 979* 79–92 (2011).

[6] Adjatin, A. et al. Proximate, mineral and vitamin C composition of vegetable Gbolo (Crassocephalum Rubens (Juss Ex Jacq) S Moore and C crepidioides (Benth) S Moore) in Benin. *Int. J. Biol. Chem. Sci.* **7**(1), 319–331 (2013).

[7] Feyssa, D. H., Njoka, J. T., Asfaw, Z. & Nyangito, M. M. Wild edible fruits of importance for human nutrition in semi-arid parts of East Shewa Zone, Ethiopia: associated indigenous knowledge and implications to food security. *Pak. J. Nutr.* **10**(1), 40–50 (2011).

[8] Mensah, J. K., Okoli, R. I., Ohaju-Obodo, J. O. & Eifediyi, K. Phytochemical, nutritional and medical properties of some leafy vegetables consumed by Edo people of Nigeria. *Afr. J. Biotechnol.* **7**, 14 (2008).

[9] Mal, B. Neglected and underutilized crop genetic resources for sustainable agriculture. *Indian J. Plant. Genetic Resour.* **20**(01), 1–14 (2007).

[10] Keatinge, J. et al. Indigenous vegetables worldwide: their importance and future development. *Acta Hort.* **1102**, 1–20. 10.17660/actahortic.2015.1102.1 (2015).

[11] Vodouhe, R., Dansi, M., Avohou, H. T., Kpei, B. & Azihou, F. Plant domestication and its contributions to in situ conservation of genetic resources in Benin. *Int. J. Biodivers. Conserv.* (2011). 10.1100/2012/176939 PMC3366210 22693431

[12] Ocvirk, S., Kistler, M., Khan, S., Talukder, S. H. & Hauner, H. Traditional medicinal plants used for the treatment of diabetes in rural and urban areas of Dhaka, Bangladesh—an ethnobotanical survey. *J. Ethnobiol. Ethnomed.* **9**(43), 2–8 (2013). 23800215 10.1186/1746-4269-9-43 PMC3702453

[13] Siddique, K. H., Li, X. & Gruber, K. Rediscovering Asia’s forgotten crops to fight chronic and hidden hunger. *Nat. Plants* **7**(2), 116–122 (2021). 33594263 10.1038/s41477-021-00850-z

[14] Hossain, S. M. M. Horticultural research in Bangladesh. In *Paper presented in World Conference on Horticultural Research, Rome, Italy* 12 (1998).

[15] Pandey, S. & Gupta, R. K. Screening of nutritional, phytochemical, antioxidant and antibacterial activity of *Chenopodium album* (Bathua). *J. Pharmacogn. Phytochem.* **3**(3), 01–09 (2014).

[16] Linney, G. Coccinia grandis (L.) Voight: a new cucurbitaceous weed in Hawaii. *Hawaii. Bot. Soc. Newsl.* **25**(1), 3–5 (1986).

[17] Sarker, U. & Oba, S. Nutraceuticals, antioxidant pigments, and phytochemicals in the leaves of Amaranthus spinosus and Amaranthus viridis weedy species. *Sci. Rep.* **9**(1), 20413 (2019). 31892700 10.1038/s41598-019-50977-5 PMC6938518

[18] Raja, R. R. et al. *Moringa oleifera*—an overview. *RA J. Appl. Res.* **2**(9), 620–624 (2016).

[19] Kashyap, P. et al. Recent advances in drumstick *Moringa oleifera*) leaves bioactive compounds: composition, health benefits, bioaccessibility, and dietary applications. *Antioxidants* **11**(2), 402 (2022). 10.3390/antiox11020402 PMC8869219 35204283

[20] Nasir, N. *Nutritive Value of Iindigenous Leafy Vegetables of Khulna* (Springer, 2019).

[21] Macneish, R. S. Speculation about how and why food production and village life developed in the Tehuacan Valley, Mexico. *Archaeology* **24**(4), 307–315 (1971).

[22] Raus, T. Taxonomic, nomenclatural and floristic review of Amaranthaceae of Greece and neighbouring countries. *Willdenowia* **52**(3), 335–357 (2022).

[23] Schmitzer, V., Veberic, R. & Stampar, F. Prohexadione-Ca application modifies flavonoid composition and color characteristics of rose (Rosa Hybrida L.) flowers. *Sci. Hort.* **146**, 14–20 (2012).

[24] Akter, J. et al. Colour, nutritional composition and antioxidant properties of dehydrated carrot (Daucus carota var. Sativus) using solar drying techniques and pretreatments. *Heliyon* **10**, 2. 10.1016/j.heliyon.2024.e24165 (2024). 10.1016/j.heliyon.2024.e24165 PMC10825429 38293496

[25] Hassan, J. et al. Optimizing textile dyeing wastewater for tomato irrigation through physiochemical, plant nutrient uses and pollution load index of irrigated soil. *Sci. Rep.**12*(1), 10088 (2022). 10.1038/s41598-022-11558-1 PMC9203507 35710771

[26] Suborna, M. N. et al. Color, antioxidant and nutritional composition of dehydrated Country bean (Lablab purpureus) seeds using solar drying techniques and pretreatments in Bangladesh. *Heliyon* **10**(10), e30936. 10.1016/j.heliyon.2024.e30936 (2024). 38799739 10.1016/j.heliyon.2024.e30936 PMC11126844

[27] Elgailani, I. E. H., Elkareem, M. A. M. G., Noh, E., Adam, O. & Alghamdi, A. Comparison of two methods for the determination of vitamin C (ascorbic acid) in some fruits. *Am. J. Chem.* **2**(1), 1–7 (2017).

[28] Zhou, Y. et al. Classification and association analysis of Gerbera (Gerbera hybrida) flower color traits. *Front. Plant Sci.* **12**, 779288 (2022). 35145530 10.3389/fpls.2021.779288 PMC8824200

[29] Morshed, M. M., Rana, M. S., Emran, T. B., Sohel, M. D. & Kawsar, M. H. Nutritional analysis and mineral content determination of *Emilia sonchifolia* DC. *Bangladesh Pharm. J.* **24**(1), 54–60 (2021).

[30] Mohammed, S., Edna, M. & Siraj, K. The effect of traditional and improved solar drying methods on the sensory quality and nutritional composition of fruits: a case of mangoes and pineapples. *Heliyon* **6**, 6 (2020). 10.1016/j.heliyon.2020.e04163 PMC7305395 32577561

[31] Nyangena, I. O., Owino, W. O., Imathiu, S. & Ambuko, J. Effect of pretreatments prior to drying on antioxidant properties of dried mango slices. *Sci. Afr.* **6**, e00148 (2019). 10.1007/s13197-019-03857-9 PMC6675852 31413411

[32] Rashid, S. A., Rehmani, F. S., Arman, M., Ibrahim, M. & Shafique, S. Estimation of moisture content & metal ions in white flowers of Bougainvillea spectabilis and Purple flowers of *Bougainvillea glabra* in Pakistan. *Pak. J. Chem.* **1**, 190–192 (2011).

[33] Gupta, D. K., Palma, J. M. & Corpas, F. J. (eds) *Antioxidants and Antioxidant Enzymes in Higher Plants* (Springer International Publishing, 2018).

[34] Dhanani, T., Shah, S., Gajbhiye, N. A. & Kumar, S. Effect of extraction methods on yield, phytochemical constituents and antioxidant activity of *Withania somnifera*. *Arab. J. Chem.* **10**, S1193–S1199 (2017).

[35] Bhatti, M. Z., Ismail, H. & Kayani, W. K. Plant secondary metabolites: therapeutic potential and pharmacological properties. In *Secondary Metabolites-Trends and Reviews* (IntechOpen, 2022).

[36] Hanif, R., Iqbal, Z., Iqbal, M., Hanif, S. & Rasheed, M. Use of vegetables as nutritional food: role in human health. *J. Agric. Biol. Sci.* **1**(1), 18–22 (2006).

[37] Martín, J., Navas, M. J., Jiménez-Moreno, A. M. & Asuero, A. G. Anthocyanin pigments: importance, sample preparation and extraction. *Phenolic Compounds-Nat. Sourc. Import. Appl.* **2017**, 117–152 (2017).

[38] Rad, J. S., Alfatemi, S. M. H., Rad, M. S. & Iriti, M. In-vitro antioxidant and antibacterial activities of *Xanthium strumarium* L. extracts on methicillin-susceptible and methicillin-resistant Staphylococcus aureus. *Anc. Sci. Life*. **33**(2), 109 (2013). 25284944 10.4103/0257-7941.139050 PMC4171851

[39] Vimala, S. & Mohd. Ilham Adenan, M. I. A. *Malaysian Tropical Forest Medicinal Plants: A Source of Natural Antioxidants* (Springer, 1999).

[40] Ojiewo, C. The role of vegetables and legumes in assuring food, nutrition, and income security for vulnerable groups in Sub-Saharan Africa. *World Med. Health Policy**7*(3), 187–210 (2015).

[41] Andersen, O. M. & Markham, K. R. *Flavonoids: Chemistry, Biochemistry and Applications* (CRC, 2005).

[42] Okpuzar, J., Ogbunugafor, H. & Kareem, G. K. & Igwo-Ezikpe, M. N. In vitro investigation of antioxidant phenolics compounds in extract of *Senna alata*. *Res. J. Phytochem.* **3**, 68–76 (2009).

[43] Jahan, F. et al. *Amaranthus tricolor* (red amaranth), an indigenous source of nutrients, minerals, amino acids, phytochemicals,and assessment of its antibacterial activity. *J. Agric. Food Res.**10*, 100419 (2022).

[44] Nazmy, S. et al. Biochemical studies on *Moringa oleifera* leaves extract. *J. Biol. Agric. Healthc.* **6**(16), 33–42 (2016).

[45] Osawa, T. *Novel Natural Antioxidants for Utilization in Food and Biological Systems* (Post-Harvest Biochemical plant food-materials in the Tropics, 1994).

[46] Abhishek, J. Why do you see various shades of green in A garden? *ScienceABC*. https://www.scienceabc.com/nature/why-do-you-see-different-shades-of-green-in-a-garden.html (2023).

[47] Rajyalakshmi, P. et al. Total carotenoid and beta-carotene contents of forest green leafy vegetables consumed by tribals of south India. *Plant Foods Hum. Nutr.* **56**, 225–238 (2001). 11442223 10.1023/a:1011125232097

[48] Duma, M., Alsina, I., Zeipina, S., Lepse, L. & Dubova, L. Leaf vegetables as source of phytochemicals. In *9th Baltic Conference on Food Science and Technology Food for Consumer Well-Being FOODBALT 2014 Conference Proceedings. Jelgava, LLU. P* 262–265 (2014).

[49] Lakshminarayana, R., Raju, M., Krishnakantha, T. P. & Baskaran, V. Determination of major carotenoids in a few Indian leafy vegetables by high-performance liquid chromatography. *J. Agric. Food Chem.* **53**(8), 2838–2842 (2005). 15826027 10.1021/jf0481711

[50] Arasaretnam, S., Kiruthika, A. & Mahendran, T. Nutritional and mineral composition of selected green leafy vegetables. *Ceylon J. Sci.* **47**(1), 35 (2018).

[51] Raju, M., Varakumar, S., Lakshminarayana, R., Krishnakantha, T. P. & Baskaran, V. Carotenoid composition and vitamin A activity of medicinally important green leafy vegetables. *Food Chem.* **101**(4), 1598–1605 (2007).

[52] Umar, K. J., Hassan, L. G., Dangoggo, S. M., Maigandi, S. A. & Sani, N. A. Nutritional and anti-nutritional profile of spiny amaranth (*Amaranthus viridis* Linn). *Studia Univ. Vasile Goldis Seria Stiintele Vietii (Life Sci. Ser.)* **21**, 4 (2011).

[53] Satter, M. M. A. et al. Nutritional quality and safety aspects of wild vegetables consume in Bangladesh. *Asian Pac. J. Trop. Biomed.* **6**(2), 125–131 (2016).

[54] Afolayan, A. J. & Jimoh, F. O. Nutritional quality of some wild leafy vegetables in South Africa. *Int. J. Food Sci. Nutr.* **60**(5), 424–431 (2009). 19037794 10.1080/09637480701777928

[55] Dasgupta, N. & De, B. Antioxidant activity of some leafy vegetables of India: a comparative study. *Food Chem.* **101**(2), 471–474 (2007).

