# Supplementary figures and images for: Nutritional composition, bioactive compounds and antioxidant potentiality of some indigenous vegetables consumed in Bangladesh
Source: Sci Rep. 2024 Nov 12;14:27699. doi: 10.1038/s41598-024-78625-7 (PMC11557952; doi:10.1038/s41598-024-78625-7)

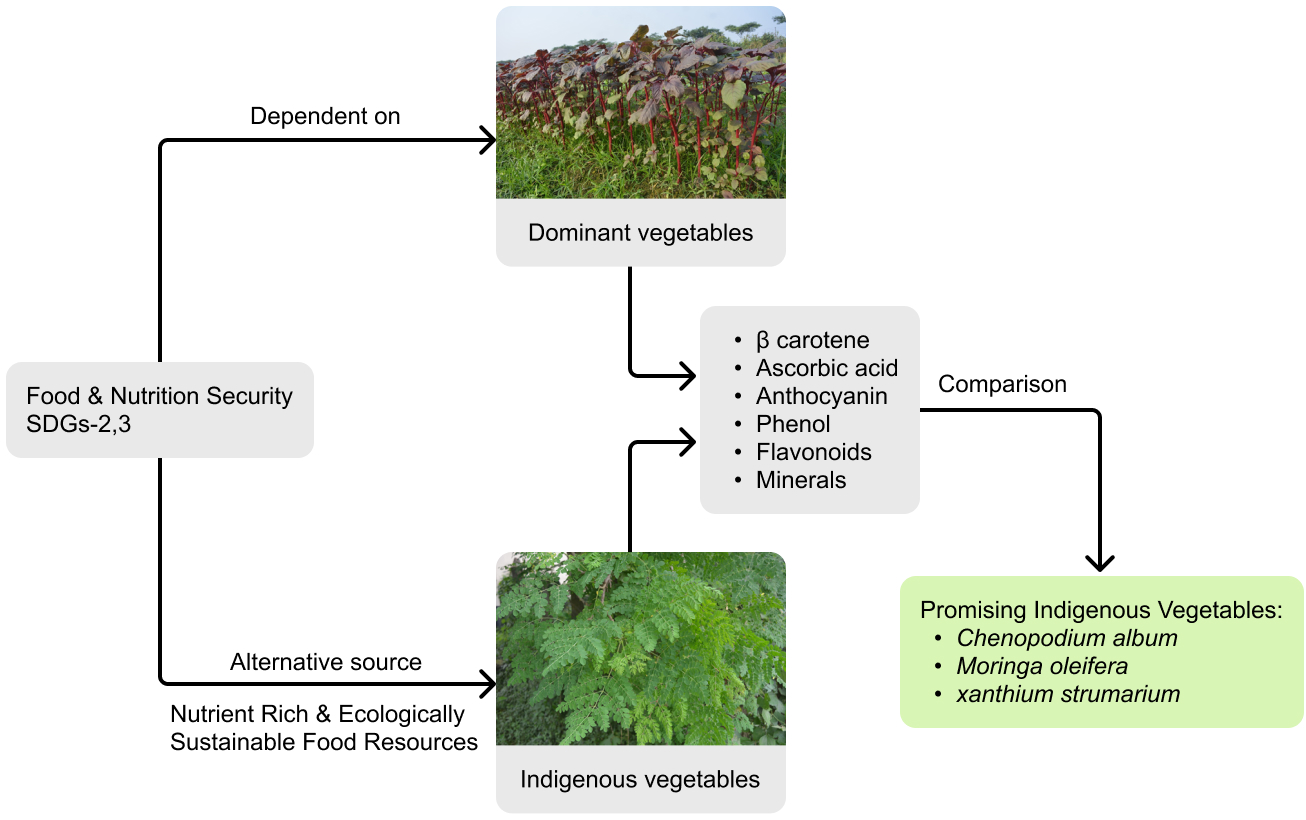

Supplement: Supplementary file 1 — Supplementary Information 1. [file 41598_2024_78625_MOESM1_ESM.jpg]
